# Two-Photon Polymerization of 2.5D and 3D Microstructures Fostering a Ramified Resting Phenotype in Primary Microglia

**DOI:** 10.3389/fbioe.2022.926642

**Published:** 2022-07-22

**Authors:** Ahmed Sharaf, Brian Roos, Raissa Timmerman, Gert-Jan Kremers, Jeffrey John Bajramovic, Angelo Accardo

**Affiliations:** ^1^ Department of Precision and Microsystems Engineering, Delft University of Technology, Delft, Netherlands; ^2^ Alternatives Unit, Biomedical Primate Research Centre, Rijswijk, Netherlands; ^3^ Erasmus Optical Imaging Centre, Erasmus MC, Rotterdam, Netherlands

**Keywords:** two-photon polymerization, micro-pillars, nano-pillars, 3D scaffold, effective shear modulus, primary microglia

## Abstract

Microglia are the resident macrophages of the central nervous system and contribute to maintaining brain’s homeostasis. Current 2D “petri-dish” *in vitro* cell culturing platforms employed for microglia, are unrepresentative of the softness or topography of native brain tissue. This often contributes to changes in microglial morphology, exhibiting an amoeboid phenotype that considerably differs from the homeostatic ramified phenotype in healthy brain tissue. To overcome this problem, multi-scale engineered polymeric microenvironments are developed and tested for the first time with primary microglia derived from adult rhesus macaques. In particular, biomimetic 2.5D micro- and nano-pillar arrays (diameters = 0.29–1.06 µm), featuring low effective shear moduli (0.25–14.63 MPa), and 3D micro-cages (volume = 24 × 24 × 24 to 49 × 49 × 49 μm^3^) with and without micro- and nano-pillar decorations (pillar diameters = 0.24–1 µm) were fabricated using two-photon polymerization (2PP). Compared to microglia cultured on flat substrates, cells growing on the pillar arrays exhibit an increased expression of the ramified phenotype and a higher number of primary branches per ramified cell. The interaction between the cells and the micro-pillar-decorated cages enables a more homogenous 3D cell colonization compared to the undecorated ones. The results pave the way for the development of improved primary microglia *in vitro* models to study these cells in both healthy and diseased conditions.

## Graphical Abstract

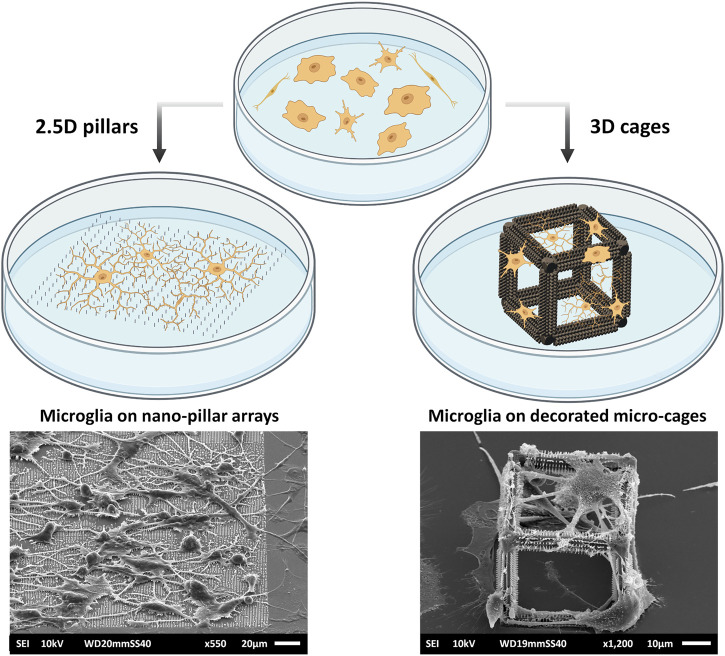

## 1 Introduction

Cell culture methods used nowadays for *in vitro* studies, mostly use stiff Petri dishes featuring a 2D planar geometry. This type of environment does not recapitulate the properties of the extracellular matrix (ECM) within which cells reside in the human body (i.e., in an *in vivo* environment). This is especially true for cells in the central nervous system (CNS) which includes the brain and spinal cord. The ECM of the CNS is made up of a complex 3D network of multiple macromolecules that are entangled together. Features in the ECM can be as small as 9 nm (Kim et al., 2018). In terms of stiffness, brain tissue is among the softest tissues in the body with a Young’s modulus ranging from 0.1 to 1 kPa (Lu et al., 2006). In comparison, the Young’s moduli of traditional Petri dishes made of polystyrene or glass are roughly 3 GPa or 70 GPa, respectively (Espinosa-Hoyos et al., 2018; Fekete et al., 2018). Cells in the CNS largely fall under one of two categories, neurons and glia (or glial cells) (Zuchero and Barres, 2015). Microglia, in particular, are the macrophages of the CNS, and play a key role in innate immune responses (Saijo and Glass, 2011; Timmerman et al., 2018). These cells have a vital role in tissue repair as they contribute to resistance to infections and maintain the homeostasis (i.e., normal function and stable state) of the brain by clearing foreign bodies and cellular debris, such as damaged axons and dead neurons through phagocytosis, which is translated from Latin as “cell eating” (Jin and Yamashita, 2016; Hickman et al., 2018; Uribe-Querol and Rosales, 2020).

When cultured *in vitro*, microglia show different morphologies (Jeong et al., 2013) and gene regulation (Lund et al., 2006) from their *in vivo* counterparts. In terms of morphology, the ramified phenotype, which is a characteristic of homeostatic microglia (Smith et al., 2012; Torres-Platas et al., 2014, p.), is rarely seen *in vitro* since primary microglia mostly show either round, globular-like or flat, amoeboid-like, morphologies (Jeong et al., 2013). This shows a stark difference from the abundance of ramified microglia, with their small somas and long branches (Torres-Platas et al., 2014), that can be found in a homeostatic brain (Smith et al., 2012). Due to these discrepancies in terms of morphology and behavior and due to the major role played by microglia in brain homeostasis and disease, biomimetic, *in vitro* models must be devised to study these cells in a physiologically relevant context.

There are multiple examples of studies focusing on using micro- and nanotopographies to alter the morphology and phenotypic expression of microglia (Persheyev et al., 2011; Luu et al., 2015; Pires et al., 2015; Kim et al., 2018). These features are sensed by microglia via lamellipodia and filopodia in a process known as mechanosensing (Small et al., 2002; Chen et al., 2017; Choi, 2020). The results of these studies in general indicate that micro- and nanotopographies may increase microglial polarization and the production of anti-inflammatory cytokines, thereby inducing a more homeostatic behavior. The reason is hypothesized to be the resemblance of such features to the native ECM in the CNS (Kim et al., 2018). For instance, BV-2 microglial cells (murine cell line) were shown to be affected by nano- and micro-patterned structures on treated silicon surface. Increasing the grain size of the structures promoted cell elongation compared to a flat and untreated surface (Persheyev et al., 2011).

Another critical factor in affecting cell behavior is the stiffness of a substrate. Cells have the ability to sense the mechanical strength of their surrounding environment. Studies performed on macrophages have shown the various and inconsistent effects of substrate stiffness on the morphology and cytokine expression of these cells (Sridharan et al., 2019; Blaschke et al., 2020; Chen et al., 2020; Dudiki et al., 2020). While some results show the polarization of microglia on softer substrates (Blaschke et al., 2020), others show the opposite (Dudiki et al., 2020).

Instead of using a soft material like hydrogels, a rather innovative way of obtaining a surface with relatively low stiffness is by utilizing the geometrical properties of large pillar arrays of stiff materials to affect the effective shear modulus of the array. The effective shear modulus of a pillar array can be explained as the shear modulus experienced by the cell as it moves over the array. Pillars with large aspect ratios result in arrays with lower effective shear moduli since long and slender pillars bend more readily in the direction of the force exerted by a cell. This reduction in the effective shear modulus was shown to significantly enhance the differentiation of human embryonic stem cells into endoderm cells (Rasmussen et al., 2016). The main advantage of such method lies in the relative ease of fabrication of pillar arrays made of stiff materials while still being able to achieve a stiffness low enough to approach the one of brain tissue. In addition, these patterns provide a unique discrete type of topography resembling that of the native ECM (Kim et al., 2018).

Fabrication of micro- and nanotopographies as well as 3D biomimetic scaffolds (Fan et al., 2019) for *in vitro* studies can be performed via multiple techniques such as emulsion templating (Riesco et al., 2019), electrospinning (Pires et al., 2015; Wissing et al., 2019; Venugopal et al., 2021), stereolithography (Gauvin et al., 2012), and two-photon polymerization (2PP) (Lemma et al., 2019; Varapnickas and Malinauskas, 2020) to name a few. While each of these fabrication methods has its own advantages, 2PP especially stands out due to its extremely high resolution (∼50–200 nm) (Malinauskas et al., 2010; Emons et al., 2012), ability to fabricate complex 3D geometries using computer-aided design (CAD) models, and high reproducibility. The most significant limitation of 2PP is the relatively long printing time hindering the upscaling of the technology (Moroni et al., 2018). Multiple polymeric, hydrogel or composite materials were employed in combination with 2PP to fabricate micro- and nano-patterns as well as 3D structures for *in vitro* cellular studies involving neuroblastoma, glioblastoma, prostate cancer, murine cerebellar granule, chondrocytes, macrophages, neuronal and stem cells (Marino et al., 2013; Accardo et al., 2017, 2018; Turunen et al., 2017; Maciulaitis et al., 2019; Fendler et al., 2019; Babi et al., 2021; Bertels et al., 2021; Maciulaitis et al., 2021; Nouri-Goushki et al., 2021; Akolawala et al., 2022; Costa et al., 2022).

To date, there has been no study exploring the effect of topography and stiffness on primary microglia derived from primates. Further, in terms of biomimicry of the native 3D environment of microglia, to the best of our knowledge, there has been no study employing free standing 3D structures including micro- and nanometric features as an added step of attaining resemblance to the *in vivo* 3D environment. To address these gaps in the field, in the current study, we tackle the research question of whether it is possible to foster the ramified resting phenotype in rhesus macaque primary microglia cells by culturing them on polymeric micro- and nano-pillar arrays, fabricated by 2PP, and exploiting the geometrical and mechanical cues of these patterns. We hypothesize that by approaching the topography and the stiffness of the ECM in the CNS (where the brain features, respectively, protein fibers of tens to hundreds of nm diameter (Kim et al., 2018) and a Young’s modulus of 0.1–1 kPa), a biomimetic *in vitro* microenvironment can be created, thereby inducing the expression of the ramified phenotype of microglia, characteristic of a healthy brain. In addition, we investigate the effect of coating the microstructures with laminin since it is one of the most abundant proteins in the ECM of the brain (Kim et al., 2018) and has been shown to affect the morphology of microglia cultured *in vitro* (Chamak and Mallat, 1991; Tam et al., 2016). In an attempt to take our investigation further, we use 2PP to fabricate 3D micro-cages decorated with micro- and nano-pillars to provide the cells with a well-structured three-dimensional environment, and we assess their effect on the phenotypic expression of microglia.

## 2 Materials and Methods

### 2.1 Design of 2D Pedestals and 2.5D Micro- and Nano-Pillar Arrays

All the structures employed for cell culturing were designed using SOLIDWORKS 2019 (DASSAULT SYSTEMES), a 3D CAD modelling software. Three types of structures were designed, herein referred to as 2D, 2.5D, and 3D. The 2D structure was a pedestal (l × w × h = 130 × 130 × 20 μm^3^) mainly used to test the viability of cells on the material employed for printing (Supplementary Figure S1A). Pedestals were designed to cover a total area of 400 × 400 μm^2^ on each substrate by printing arrays of 3 × 3 pedestals with an interspacing of 5 µm. The 2.5D category included the micro-pillar (MP) and nano-pillar (NP) arrays (Supplementary Figure S1B). All pillar arrays were designed to cover a total area of 500 × 500 μm^2^. The micro-pillars were designed to have a diameter (d) of 1 μm, a height (h) of 2.5 µm, and an inter-pillar spacing (p) of 1 µm (edge-to-edge). Two versions of the nanopillar arrays were designed (NP1 and NP2). NP1 arrays were designed to be directly printed on the substrate while NP2 ones were designed to be printed on top of a pedestal of 500 × 500 × 5 μm^3^ dimensions (l × w × h) to increase the stability of the pillars. The designed dimensions of the pillars in both NP1 and NP2 arrays, were the same (d = 0.2 µm, h = 2.5 µm, *p* = 1 µm).

### 2.2 Design of the 3D Cages

Concerning the 3D category, five structures in total were designed. The structures were cuboidal micro-cages of two different sizes. They were designed to either have no decoration, a micro-pillar decoration, or a nano-pillar decoration on their beams (Supplementary Figure S1C). The small cage (SC) and micro-pillar-decorated small cage (SC-MP) structures were designed to have a volume of 25 × 25 × 25 μm^3^ (L × W × H) and to be printed in arrays of 7 × 7 cages in order to cover a total area of 500 × 500 μm^2^. Concerning the SC-MP, the pillar diameter and height were designed to be 1 and 2.5 µm, respectively. The angular spacing (θ) was 30°, and the lateral pillar spacing (δ) was 1 µm. On the other hand, the big cage (BC), micro-pillar-decorated big cage (BC-MP), and nano-pillar-decorated big cage (BC-NP) structures were designed to have a volume of 50 × 50 × 50 μm^3^ (L × W × H) and to be printed in arrays of 5 × 5 cages in order to cover a total area of 550 × 550 μm^2^. All the beams of the cages, whether small or big, were cylindrical in shape (D = 5 µm). The micro-pillars on the BC-MP were designed to have a diameter of 1 µm while nano-pillars on the BC-NP structures had a diameter of 0.2 µm. All pillars were designed to have a height of 2.5 µm, an angular spacing (θ) of 30°, and a lateral pillar spacing (δ) of 1 µm.

### 2.3 Preparation of the Substrate

**FIGURE 1:**
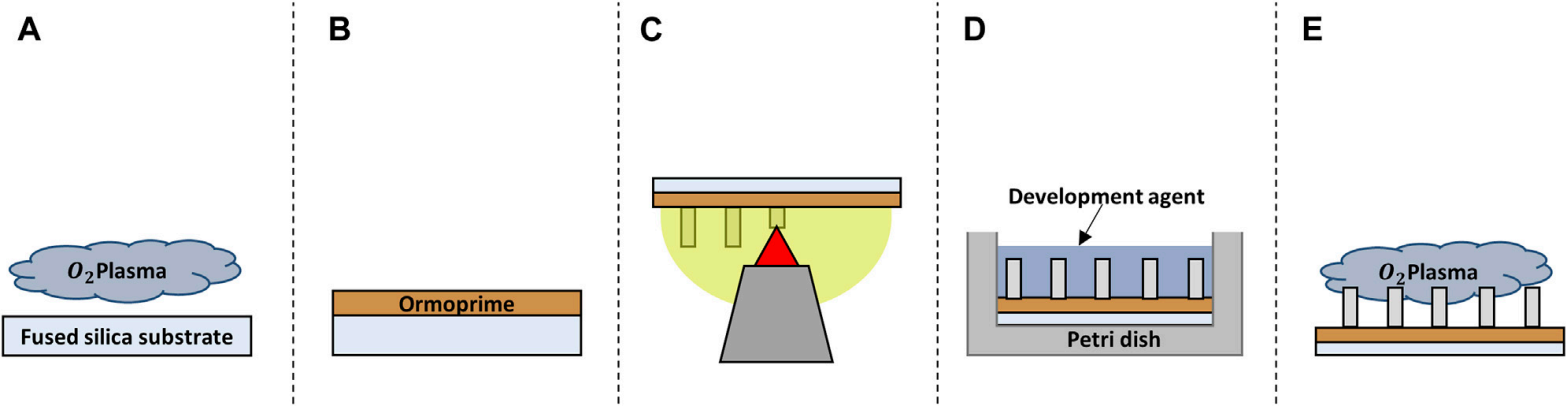
Schematic representation of the fabrication process of the structures. **(A)** Oxygen plasma cleaning of the substrate. **(B)** Spin coating of the adhesion promoter Ormoprime^®^ 08. **(C)** 2PP printing of the structures. **(D)** Chemical development of the structure. **(E)** Oxygen plasma functionalization of the polymer surface.

All structures were printed on 25 × 25 × 0.7 mm^3^ (l × w × h) fused silica substrates (Nanoscribe GmbH & Co. KG) and, unless otherwise mentioned, all chemicals were purchased from Sigma-Aldrich. Before printing, the substrates (Young’s modulus ≈72 GPa (Torres-Torres et al., 2010)) were first cleaned with acetone and iso-propanol using a lint-free wipe and then dried with an air-gun. The samples were then further cleaned and activated using a Diener oxygen plasma cleaner for 5 min at 80 W with a gas flow rate of 5 cm^3^/min (Figure 1A). The substrates were coated with OrmoPrime^®^ 08 (Microresist Technology GmbH) to increase adhesion of the structures. Multiple droplets of OrmoPrime^®^ 08 were cast and spincoated (spin-coating speed = 4,000 rpm, time = 60 s, acceleration = 1,000 rpm/s) on top of the substrate to achieve a coating thickness of 130 ± 15 nm (Figure 1B). The substrates were then baked on a hot plate for 5 min at 150°C to harden the coating. Finally, the substrates were placed in the sample holder, and one droplet of the negative tone IP-Dip photoresist (Nanoscribe GmbH & Co. KG) was deposited in the middle of the substrate before printing.

### 2.4 Printing Parameters

The structures were printed employing the Nanoscribe Photonic Professional GT+ setup, with a femtosecond pulsed laser working at a wavelength of 780 nm (Nanoscribe GmbH & Co. KG). A 63× objective with a numerical aperture of 1.4 was operated in Dip-in Laser Lithography (DiLL) mode in order to polymerize the IP-Dip photoresist (Figure 1C shows for simplicity only the pillars structures). The 3D models of the structures were imported into Describe (the proprietary software of Nanoscribe GmbH & Co. KG) as (.stl) files where they were split into horizontal lines (hatching lines) and vertical layers (slices) since printing takes place in a layer-by-layer fashion in the vertical direction where each layer is made up of multiple horizontal lines. Hatching and slicing distances for all structures (2D, 2.5D, and 3D) were 0.2 and 0.25 µm respectively except for the nano-pillars on the BC-NP scaffolds where the hatching and slicing were 0.1 and 0.2 µm respectively. Afterwards, the printing parameters (i.e., laser power and scanning speed) were optimized for each structure. The pedestals were printed at a laser power of 42.5 mW (85% of a maximum power of 50 mW) and scanning speed of 60 mm/s. There were two versions of the micro-pillar arrays depending on the printing parameters (MP1 and MP2). The MP1 arrays were printed at a laser power of 42.5 mW and a scanning speed of 60 mm/s while the MP2 ones were printed at a laser power of 35 mW (70% of a maximum power of 50 mW) and a scanning speed of 30 mm/s. Similarly, the laser power and scanning speeds employed to manufacture NP1 and NP2 arrays were 42.5 mW and 60 mm/s, and 35 mW and 30 mm/s respectively. The printing parameters of all 3D cages were 35 mW and 30 mm/s. The corresponding light intensity values (Skliutas et al., 2021) are summarized in Supplementary Table S1 together with additional parameters of the employed laser source.

### 2.5 Sample Development and Post-processing

Once the printing process was completed, the substrates with polymerized structures were carefully placed horizontally in a borosilicate Petri dish filled with propylene glycol methyl ether acetate (PGMEA) for 25 min to dissolve the unpolymerized resin. This was followed by submersion in a Petri dish with iso-propanol for 5 min to rinse off the excess PGMEA. The samples were then submersed for 30 s in Novec™ 7100 Engineered Fluid, which has lower surface energy than iso-propanol resulting in a decrease of collapsed nano-pillars as a result of mechanical stresses caused by wet-to-dry transitions (Figure 1D). Post-processing of the IP-Dip structures was performed by plasma activation using the Diener oxygen plasma cleaner for 20 s at 80 W with a gas flow rate of 5 cm^3^/min (Figure 1E). This would additionally activate the surface of the polymeric structures to increase the adhesion of the biochemical coating and/or the cells to the structures.

### 2.6 Mechanical Characterization of the Structures

In order to assess the Young’s modulus of the material, a series of four compression tests were performed using the FEMTOTOOLS nanomechanical testing system FT-NMT03 (Supplementary Figures S2A,B). The 2D pedestal structure was used for this test. The compression test was performed at a displacement of 10% of the height of the structure. The employed probe was a flat silicon one, model FT-S200,000, had a tip size of 50 × 50 μm^2^ and a force range of 200,000 ± 0.5 µN. To confirm that the Young’s modulus of the material does not change significantly at the nanoscale, an Atomic Force Microscope (Bruker JPK Nanowizard 4) was employed to measure the mechanical properties of a single nano-pillar since the probes of the FT-NMT03 were too large in size to perform such a measurement. This was accomplished with a pillar bending test, using the cantilever tip to bend the nano-pillars (cantilever stiffness 14.4 N/m) (Supplementary Figures S2C,D), resulting in a force versus displacement diagram from which the spring constant of the structure was derived (Angeloni et al., 2021). Using beam deflection theory, and assuming a cylindrical shaped cantilever beam with an external force at the tip, the Young’s modulus was obtained. The calculation of the effective shear moduli of the micro- and nano-pillar arrays was achieved by using Eq. 1 (Rasmussen et al., 2016).

$$\bar{G}=\frac{3}{16}{\left(\frac{D}{L}\right)}^{2}\mathrm{fE}$$ (1)

Where $\bar{G}$ is the effective shear modulus, *D* and *L* are the diameter and height of the pillar respectively, *f* is the surface coverage (surface area covered by pillars per total surface area of the array), and *E* is the Young’s modulus of the bulk material.

### 2.7 Sample Sterilization

The substrates with or without structures were transferred to a 6-well plate. Within the 6-well plate, the substrates were then washed twice for 5 min with a large volume (∼4 ml/well) of 70% ethanol. Finally, the substrates were washed five times for 1 min with sterile demi-water or phosphate buffer saline (PBS).

### 2.8 Laminin Coating of the Substrates

Some samples were coated with laminin to observe the effect of a biochemical coating on the phenotypic expression of microglia. To coat the samples with laminin, 10 mg/ml working solution of laminin was prepared in Dulbecco’s Modified Eagle Medium/Nutrient Mixture F-12 (DMEM/F-12) (Gibco). Then the solution was cast to cover the entire surface of the fused silica substrate (∼500 ml/substrate). For uncoated substrates, DMEM/F-12 was cast to cover the entire surface of the fused silica substrate (∼500 ml/substrate). The substrates were then incubated at 37°C and 5% CO2 for 2 h. The freshly coated substrates were then used for further experiments.

### 2.9 Primary Microglia Cell Isolation

Primary microglia were derived from isolated brain tissue (white matter) of adult rhesus macaque (*Macaca mulatta*) donors that were free from neurological diseases. All animals were outbred at the breeding colony of the Biomedical Primate Research Centre (BPRC). The BPRC has been recognized by the Dutch Ministry of Agriculture, Nature and Food Quality for performing animal experiments under the veterinary control number 7962. No animals were sacrificed for the exclusive purpose of the initiation of microglia cell cultures. Better use of experimental animals contributes to the priority 3Rs program of the BPRC. There were 6 donors in total with various ages and genders. The overview of donors is reported in the Supplementary Table S2.

Microglia isolations were initiated from cubes (∼4.5 g) of frontal subcortical white matter tissue that were depleted of meninges and blood vessels manually. The tissue was chopped into cubes of less than 2 mm^2^ by using gentle MACS™ C tubes (Miltenyi Biotec, Bergisch Gladbach, Germany) and then incubated at 37°C for 20 min in PBS containing 0.25% (w/v) trypsin (Gibco Life Technologies, Bleiswijk, the Netherlands), 1 mg/ml bovine pancreatic DNAse I (Sigma-Aldrich, Saint Louis, MO) and mixed every 5 min. The supernatant was discarded (no centrifugation), the pellet was washed and passed over a 100 mm nylon cell strainer (Falcon; Becton Dickinson Labware Europe) and centrifuged for 7 min at 524 g. The pellet was re-suspended in 22% (v/v) Percoll, 37 mM NaCl and 75% (v/v) myelin gradient buffer (5.6 mM NaH_2_PO_4_, 20 mM Na_2_HPO_4_, 137 mM NaCl, 5.3 mM KCl, 11 mM glucose, 3 mM bovine serum albumin (BSA) Fraction V, pH 7.4). A layer of myelin gradient buffer was added on top, and this gradient was centrifuged at 1,561 g for 25 min (minimal brake). The pellet was washed and centrifuged for 7 min at 524 g.

### 2.10 Microglia Cell Culture

Primary microglia cells were plated at a density of 50,000 cells/cm^2^ on either uncoated or laminin coated substrates with or without structures in serum medium (SM) comprised of 1:1 v/v DMEM (high glucose)/HAM F10 Nutrient mixture (Gibco) supplemented with 10% v/v heat-inactivated FBS (TICO Europe, Amstelveen, the Netherlands), 2 mM glutamax, 50 units/ml penicillin and 50 mg/ml streptomycin (all from Gibco). After overnight incubation at 37°C in a humidified atmosphere containing 5% CO_2_, the unattached cells and debris were removed by washing with PBS twice and replaced by fresh SM medium supplemented with 20 ng/ml (≥4 units/ml) M-CSF (PeproTech, London, United Kingdom). At day 4, cells were washed twice with PBS and replaced by serum-free microglial (SFM) culture medium comprised of DMEM/F12 (Gibco) supplemented with 0.5 mM glutamax, 50 units/ml penicillin, 50 mg/ml streptomycin, 5 mg/ml N-acetyl cysteine, 5 mg/ml insulin, 100 mg/ml apo-transferrin, 100 ng/ml sodium selenite, 20 ng/ml (≥4 units/ml) M-CSF, 12.5 ng/ml TGF-β (Miltenyi Biotec, Bergisch Gladbach, Germany), 1.5 mg/ml ovine wool cholesterol (Avanti Polar Lipids, Alabaster, AL), 1 mg/ml heparan sulfate (Galen Laboratory Supplies, North Haven, CT), 0.1 mg/ml oleic acid (Cayman Chemical, Ann Arbor, MI), 1 ng/ml gondoic acid (Cayman Chemical). All cells were kept in culture for a total of 15 days without passaging. From day 4, half of the medium was replaced by a fresh SM medium containing new growth factors every 2–3 days.

### 2.11 Scanning Electron Microscope Imaging

To prepare cells for scanning electron microscope (SEM) imaging, a fixation protocol was carried out. First, the medium was removed and the cells were washed twice with PBS (Gibco). Second, the cells were fixed with 2% paraformaldehyde (PFA) (Affymetrix, Santa Clara, CA) in PBS for 30 min at room temperature. The PFA was removed and the cells were washed with PBS twice. The PFA fixated samples were then post-fixed with 2% glutaraldehyde (GA) (Agar Scientific, Stansted, United Kingdom) in PBS for 2 h. Then the fixative was removed and the samples were dehydrated in distilled water for 2 × 5 min, followed by 50% ethanol in distilled water for 15 min, 70% ethanol in distilled water for 20 min, and lastly in 96% ethanol in distilled water for 20 min. The ethanol was then removed from the sample and hexamethyldisilazane (HMDS) was used for further drying to reduce membrane rupture of the cells. Firstly, two parts of 96% ethanol were used on one part HMDS for 15 min. Secondly, one part of 96% ethanol on one part HMDS for 15 min. Thirdly, one part of 96% ethanol on two parts HMDS for 15 min. Pure HMDS was then used for 20 min, twice. Lastly, the HMDS was removed and the sample was air-dried for 2 h.

In order to visualize the structures and the morphology of the cells, SEM imaging was carried out using a JEOL JSM-6010LA SEM (JEOL (Europe) BV) in high-vacuum with an accelerating voltage of 10 kV. Prior to imaging the samples, they were sputtered with a nanometric layer of gold using a JEOL JFC-1300 auto-fine sputter coater at a current of 20 mA for a duration of 30 s. The samples were roughly 25 mm away from the gold source. An additional sputtering step at a 45° angle was performed in order to ensure a homogeneous metal coating also along sidewalls.

### 2.12 Immunofluorescence and Confocal Imaging

The cells grown on substrates were fixed for 30 min at room temperature in 2% PFA, washed with PBS and PBS +0.02% Tween20 respectively, and a-specific binding was blocked by incubation for 30 min in PBS containing 2% normal goat serum. Samples were incubated overnight at 4°C with CX3CR1 antibody (1:400, Abcam, Cambridge, United Kingdom) in PBS containing 0.1% BSA, washed with PBS +0.02% Tween20, and incubated for 1 h at room temperature with goat anti-rabbit Alexa 647 (1:250, Jackson ImmunoResearch Laboratories, Weste Grove, PA) (red channel) in PBS containing 0.1% BSA. This antibody was used to stain and visualize the cellular membrane of microglia. After extensive washes with PBS, the substrates were incubated in 2 mM Hoechst 33342 (Thermo Fisher Scientific™, blue channel, nuclei) for 10 min, washed with PBS twice and then stored in PBS. A Leica SP5 confocal microscope (Mannheim, Germany) was used in combination with the Leica LAS-AF software. The employed excitation wavelengths were 405 nm, 488 and 633 nm in presence of a Leica Microsystems HC APO L 20.0x/1.00 W lens with a working distance of 1.95 mm and a numerical aperture of 1.0, in water dipping mode. Samples were submersed in PBS during all imaging sessions. The step size of the z-stacks was 250 nm. Both Fiji (Schindelin et al., 2012) and Imaris (Oxford Instruments) were employed for the 3D reconstructions of the z-stacks.

### 2.13 Quantitative and Statistical Analysis of Microglia

The phenotype distribution of microglia was quantified by analysing the morphology of the cells. This was achieved by using the multi-point selection tool in Fiji. To determine the degree of ramification of ramified cells (i.e., the degree of complexity and number of ramified branches that a cell has), Sholl analysis was carried out on both SEM and immunofluorescence images. First, the perimeter of the cell was traced with the Neurite tracer tool in Fiji and skeletonized. Then, using the Sholl analysis, concentric circles were drawn around the centre of the cell, with a radial increment of 5 µm. This tool measures the number of intersections that the body and branches of the cell have at each concentric circle. The number of intersections is plotted against the distance from the cell centre. A high amount of intersections translates to a high degree of ramification of the cells. The area under the curve (AUC) obtained from a Sholl plot was also used as an indication of the degree of ramification of ramified cells. The larger the area, the more ramified a cell is. Furthermore, the counting of primary branches was performed by the use of the multi-point selection tool (Fiji) on the same cells that were investigated for the Sholl analysis. No less than two donors were used for all studies performed on fused silica substrates and 2.5D structures with 1-3 samples per donor, and with a minimum of three samples per study (*n* ≥ 3).

To obtain the volumetric occupancy of the cells in the 3D cages, we assumed a volume of 60 × 60 × 60 μm^3^ around each cage and counted all cells within that volume and then divided the number of cells by said volume to obtain a cell density (cells per mm^3^). This was only performed for the BC structures since the cells completely enwrapped the SC structures leaving no room for such analysis. Lastly, cell occupancy and distribution in the bigger 3D structures were assessed and compared. The cells were counted in the fluorescent images extracted from z-stacks of the 3D cages using the multi-point selection tool in Fiji and an in-house code developed in MATLAB (MathWorks^®^). Cages were split into 15 µm thick sections (0–60 µm range) and cells were counted within each section. For 3D structure studies, one donor was used with a minimum of two samples per study. Results are reported as means and standard deviations. All means are calculated by first calculating the means for samples used with each individual donor and then averaging all results from all donors. Microsoft Excel was the main tool employed to obtain means and standard deviations.

## 3 Results and Discussion

### 3.1 Fabrication of the Micro- and Nano-Structures

**FIGURE 2:**
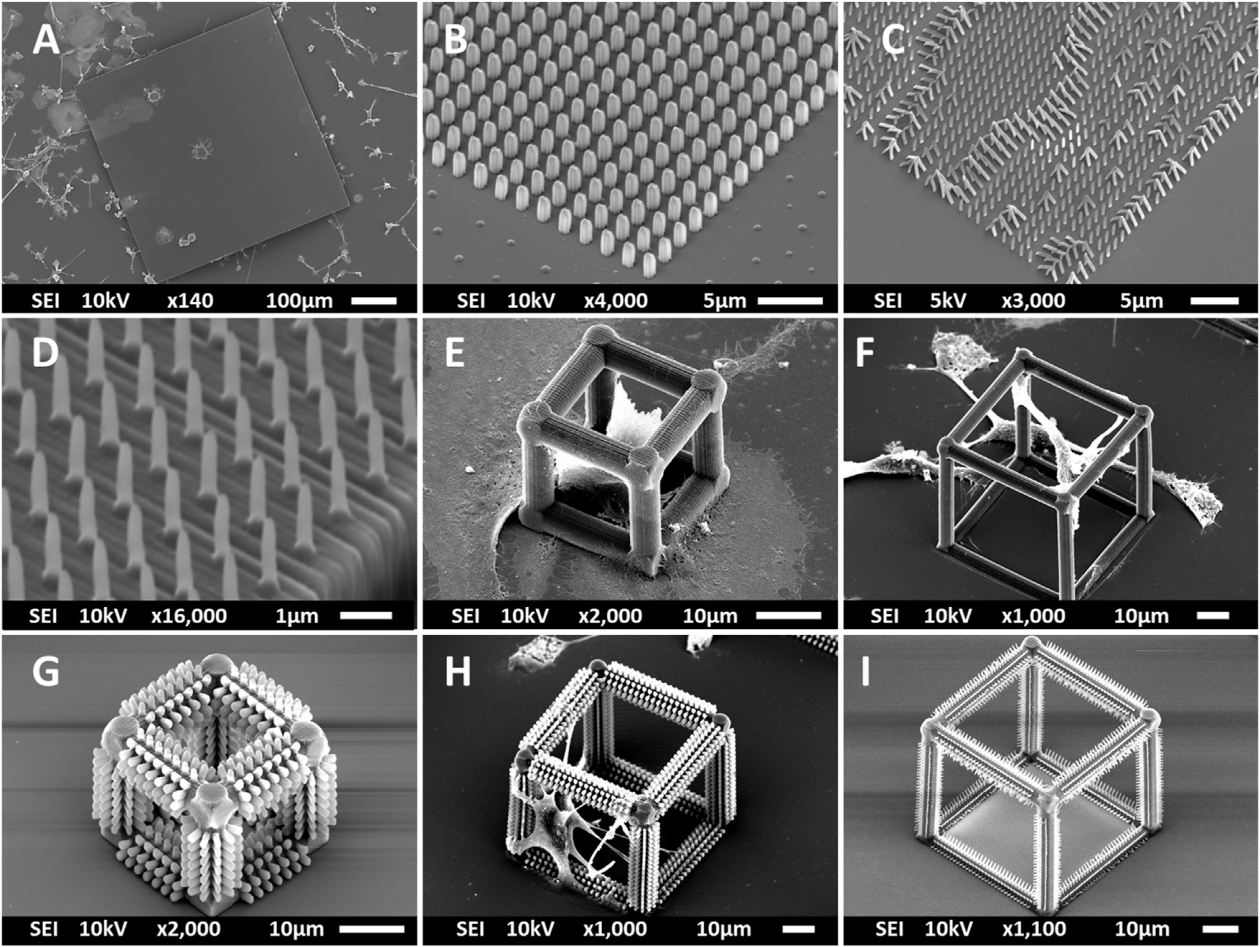
SEM images (at a 45° angle) of **(A)** the pedestal; **(B)** micro-pillar array (MP1); **(C)** nano-pillar array (NP1); **(D)** nano-pillar array (NP2). The printing of a supporting pedestal for the nano-pillars prevented the collapse of pillars after development. **(E)** Small cage (SC). **(F)** Big cage (BC). **(G)** Small cage with micro-pillar decoration (SC-MP). **(H)** Big cage with micro-pillar decoration (BC-MP). **(I)** Big cage with nano-pillar decoration (BC-NP).

Multi-scale structures were fabricated using 2PP to investigate the effect of geometry and mechanical cues on primary microglial morphology. The overview of the fabrication process is depicted in Figure 1 and detailed in Section 2. SEM images of all printed structures are shown in Figure 2. The developed structures consisted of square pedestals (Figure 2A), micro-pillar arrays (Figure 2B), nano-pillar arrays (Figures 2C,D), small cuboidal cages (Figure 2E), big cuboidal cages (Figure 2F), micro-pillar-decorated small cages (Figure 2G), micro-pillar-decorated big cages (Figure 2H), and nano-pillar-decorated big cages (Figure 2I). A higher magnification SEM micrograph of the micro- and nano-pillars decorating cuboidal cages is reported in Supplementary Figure S3.

**FIGURE 3:**
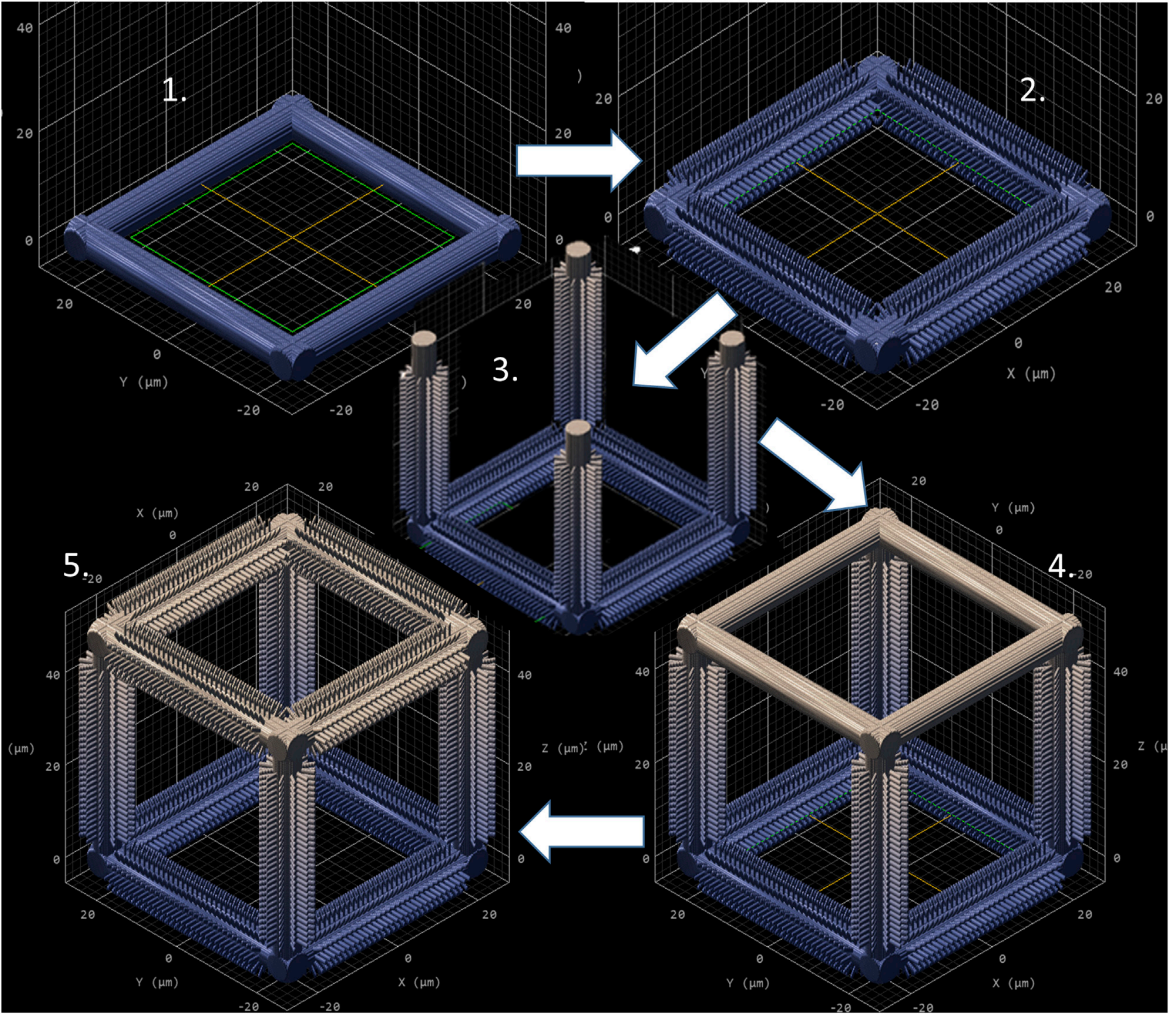
Sequential printing technique of the BC-NP, using five steps. (1) Base printed. (2) Pillars printed on the base. (3) Vertical beam with pillars printed. (4) Top printed. (5) Pillars printed on the top. CAD model generated by Describe (Nanoscribe GmbH).

As mentioned in Section 2, NP1 arrays (Figure 2C) were directly printed on the substrate while NP2 ones (Figure 2D) were printed on top of a pedestal to prevent the pillars from falling over since the forces of surface tension proved to have major detrimental effects on the stability of the pillars after the printing process. The SC and SC-MP structures were designed to have a size smaller than the cell soma, but comparable to that of the nucleus while the BC, BC-MP, and BC-NP structures were designed to have sizes larger than the cell soma. For more information regarding the design, fabrication and dimensions of the structures, the reader is referred to Section 2. Supplementary Table S3 shows the actual measured dimensions of the 2D and 2.5D structures. For the pedestal, an average of 5% difference between measured dimensions and nominal ones was noticed. For micro-pillars in the MP1 arrays, the average shrinkage in the x-y and in the z-directions was 3 and 21.2%, respectively. Pillars in the MP2 arrays, however, exhibited an average increase of 6% in measured dimensions in the *x*-*y* direction compared to the nominal ones. In the *z*-direction, there was an average shrinkage of ∼25%. For the nano-pillars, in the NP1 array, an average enlargement of 95% in the *x*-*y* direction and an average shrinkage of 24% in the *z*-direction were noticed. Pillars in the NP2 array displayed an average enlargement of 45% in the *x*-*y* direction and shrinkage of ∼27% in the *z*-direction. The difference in measured dimensions from designed ones can be attributed to the shrinkage of IP-Dip post development (Goraus et al., 2018) and the high sensitivity of sub-micrometric structures to the printing parameters as well as the hatching and slicing parameters. The main reason for this is the increase in the voxel size when using a higher laser power. For the 3D cages, a similar difference in printed dimensions was observed. Supplementary Table S4 shows the actual dimensions of the cages and their micro- or nano-pillars decorations. No major challenges were faced in printing 3D structures with the exception of the BC-NP. Since the hatching and slicing distances were different for the cage (hatching distance = 0.2 µm, slicing distance = 0.25 µm) and the nano-pillar decoration (hatching distance = 0.1 µm, slicing distance = 0.2 µm), the entire structure could not be printed directly in one run. Therefore, we proposed a method in which the beams of the cage were printed first and then the pillars were printed on top of them using multiple (.stl) files (Figure 3). This printing technique, however, required the elements of the cage to be printed separately to avoid shadowing effects caused by the top part of the cage, thereby hindering the printing of pillars on the bottom and side beams. The cage was split into three sections, the bottom (consisting of four horizontal beams), the middle (consisting of four vertical beams), and the top (consisting of four horizontal beams). Each section was printed separately and then the pillars were printed on the respective section.

### 3.2 Mechanical Characterization of the IP-Dip Structures

The Young’s modulus of IP-Dip (pedestal structure), which is known to have a dependence from writing parameters (Lemma et al., 2017), was measured by compression testing using the FEMTOTOOLS nanomechanical testing system. The Young’s modulus was determined to be 1.28 ± 0.18 GPa and 1.07 ± 0.06 GPa (*n* > 3) in presence of the writing parameter sets (42.5 mW and 60 mm/s; 35 mW and 30 mm/s) employed respectively for printing the pedestals, MP1, and NP1 arrays; the MP2, NP2 arrays, and the 3D cages. By means of a pillar bending test using an atomic force microscope (AFM), the Young’s modulus was determined to be 1.80 ± 0.18 GPa (*n* > 3). Using Eq. 1 (see Section 2), the effective shear moduli of MP1, MP2, NP1, and NP2 arrays were calculated to be 11.4, 14.63, 0.91, and 0.25 MPa respectively, therefore much lower than the intrinsic Young’s modulus of IP-Dip and closer to the one of the brain tissue.

### 3.3 Classification of Microglial Phenotypes *In Vitro*

In the *in vivo* environment of human brain white matter, there are four main phenotypes of microglia, namely ramified, primed, reactive and amoeboid (Torres-Platas et al., 2014). The ramified phenotype (also known as the “resting” state of microglia) is the most abundant in a healthy brain (Smith et al., 2012). This phenotype is characterized by multiple extensively ramified processes acting as sensors that search for chemical or physical cues indicating the presence of a foreign body or cellular debris. Ramified microglia have the smallest cell body (area ≈16 μm^2^), but the longest processes (total process length ≈430 µm). On average, the number of primary processes is five. Both primed and reactive microglia have on the other hand larger cell bodies, but fewer processes than the ramified ones. The amoeboid phenotype is the phagocytic state of microglia that isolates and then phagocytoses cellular debris or foreign bodies. This phenotype has a flat morphology, a wide cell body, and a maximum of two unbranched primary processes (Torres-Platas et al., 2014).

As already pointed out earlier, the phenotypes of microglia observed *in vitro* are substantially different from their *in vivo* counterparts, both morphologically and functionally (Chao et al., 1992; Nimmerjahn et al., 2005; Jeong et al., 2013). Therefore, it was not possible to use a conventional *in vivo* classification of microglia to describe our findings. Based on our observations of microglial morphology *in vitro*, we proposed a more relevant phenotypic classification. We included four categories within this classification, namely flat amoeboid, globular, non-amoeboid, and bi-polar (Supplementary Figure S4 depicts representative SEM images of all *in vitro* phenotypes). The flat amoeboid category includes cells with a wide soma and few to no processes. This phenotype is similar to the one found *in vivo* except that the *in vitro* phenotype is more round and spread out over the surface (diameter ≈40 µm) as compared to the *in vivo* counterpart (diameter ≈12 µm). The globular phenotype includes cells that are almost spherical in shape. Such morphology should not be confused with that of dead cells, as the cell membrane is still completely intact. The bi-polar phenotype includes rod-shaped (polarized) cells. The last category is the non-amoeboid one and it consists of cells of two phenotypes, the ramified and the non-ramified ones. Ramified microglia look morphologically like the *in vivo* counterpart in terms of ramified branching that spread in multiple directions. A cell is considered ramified if it has at least three primary branches and a branch is considered primary if its length is equal to the minimum Feret diameter of the cell soma (i.e., the smallest diameter measured across the soma in all given directions). Non-ramified microglia are cells that resemble the ramified cell in morphology as they have multiple branches but not as long or branched as the ramified phenotype. This phenotype resembles the morphology of reactive *in vivo* microglia. The reason of gathering these two phenotypes in one category is their morphological resemblance and the absence of a robust and detailed method to discriminate between truly ramified cells and ramified-like cells based only on morphology in an *in vitro* cell culture.

### 3.4 Effect of Laminin Coating on Microglial Phenotype

**FIGURE 4:**
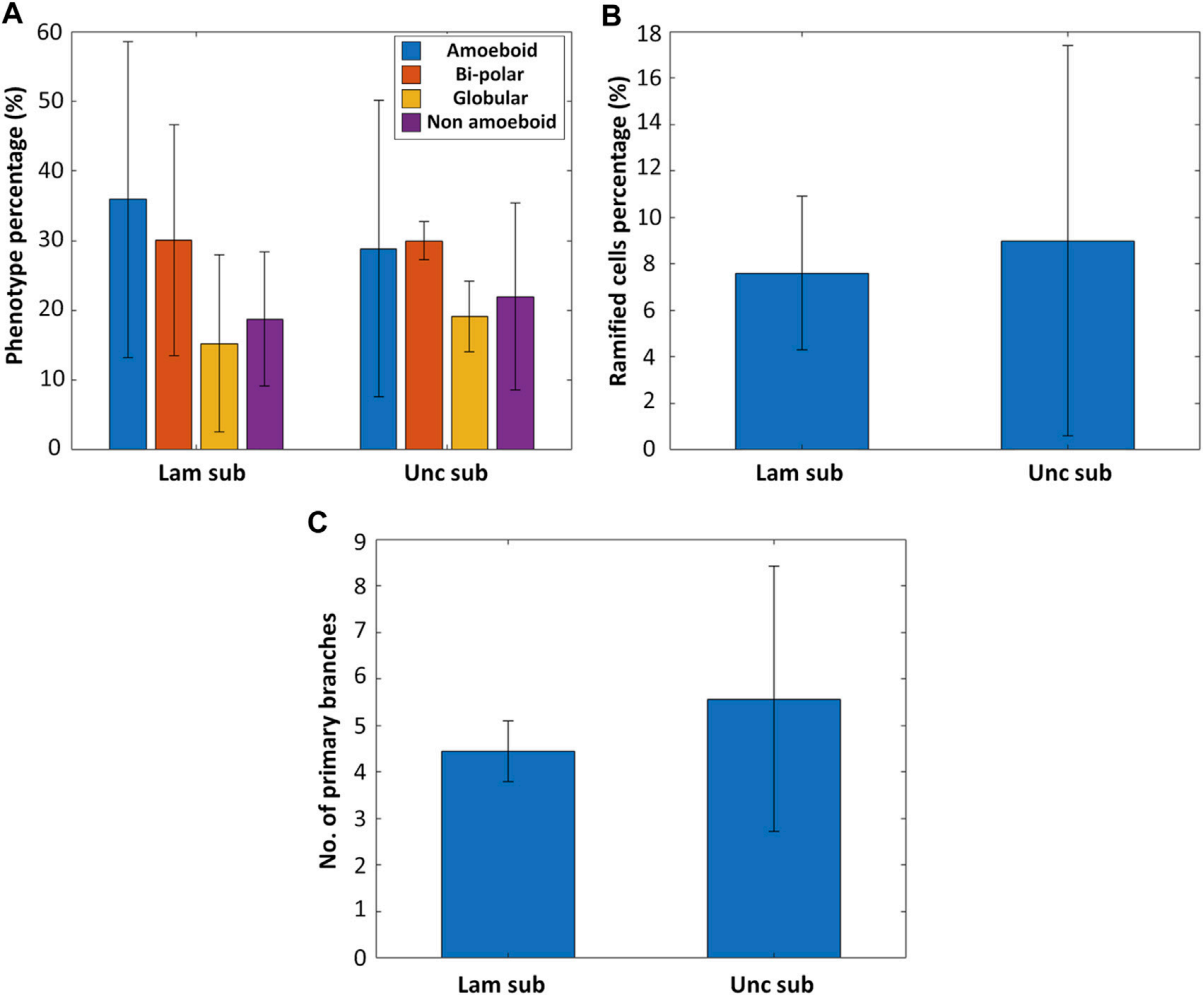
**(A)** The phenotype distribution of microglia cultured on laminin-coated fused silica substrates (Lam sub) and uncoated substrates (Unc sub) (n_samples_ = 11 and n_cells_ > 1,000 for the uncoated substrates, n_samples_ = 9 and n_cells_ > 1,000 for the laminin-coated substrates). **(B)** Percentage of ramified cells on laminin-coated versus uncoated substrates. **(C)** Number of primary branches per ramified cell for ramified microglia cultured on laminin-coated versus uncoated substrates (n_samples_ = 9 and n_ramified_cells_ > 30 for the uncoated substrates, n_samples_ = 9 and n_ramified_cells_ > 30 for the laminin-coated substrates).

Primary microglia from five rhesus macaque donors were isolated and cultured on fused silica substrates in the presence or absence of laminin coating to investigate the effect of laminin on the expression of a ramified phenotype in microglia (Supplementary Figure S5 shows representative confocal microscopy images of microglia grown on uncoated and laminin-coated substrates). For each donor, 1-3 samples were used. As depicted in Figures 4A,B, there is not a remarkable difference between laminin coated and uncoated substrates in terms of fostering a ramified phenotype (n_samples_ = 11 and n_cells_ > 1,000 for the uncoated substrates, n_samples_ = 9 and n_cells_ > 1,000 for the laminin-coated substrates). The percentage of ramified cells on the uncoated substrates was 9.0 ± 8.39% (roughly 40.9% of the non-amoeboid phenotype) while on the laminin coated ones, it was 7.61 ± 3.29% (roughly 40.5% of the non-amoeboid phenotype, Figure 4B). An additional observation was the increased complexity of the ramified microglia on the uncoated substrates shown by the higher average number of primary branches per ramified microglial cell (Figure 4C) (n_samples_ = 9 and n_ramified_cells_ > 30 for the uncoated substrates, n_samples_ = 9 and n_ramified_cells_ > 30 for the laminin-coated substrates). It is noteworthy that the high standard deviation in this data is mainly due to the large donor-to-donor variance present in an outbred colony and to the limited number of tissue donors necessary to initiate primary cell cultures, as further explained in the following section.

In general, these results indicate that a laminin coating, which can feature some inhomogeneous distribution over the substrate, does not promote the expression of a ramified phenotype, which belongs to the non-amoeboid category (as explained in Section 3.3). These results are in line with literature as it has been shown in multiple studies that laminin specifically increases a microglial amoeboid pro-inflammatory phenotype while simultaneously decreasing the expression of a ramified or resting phenotype (Chamak and Mallat, 1991; Tam et al., 2016; Pietrogrande et al., 2018). For example, Pietrogrande et al. found that culturing BV2 cells and primary murine microglia on laminin coated substrates increased the phagocytic activity and expression of pro-inflammatory cytokines as well as decreased the branching of the cells when compared to culturing on uncoated substrates. These results were corroborated by *in vivo* experiments of murine microglia (Pietrogrande et al., 2018). In a study performed by Tam et al., primary murine amoeboid microglia cultured *in vitro* were even shown to phagocytose the laminin coating on the substrate (Tam et al., 2016).

### 3.5 Effect of Micro- and Nano-Pillar Arrays on Microglial Phenotype

**FIGURE 5:**
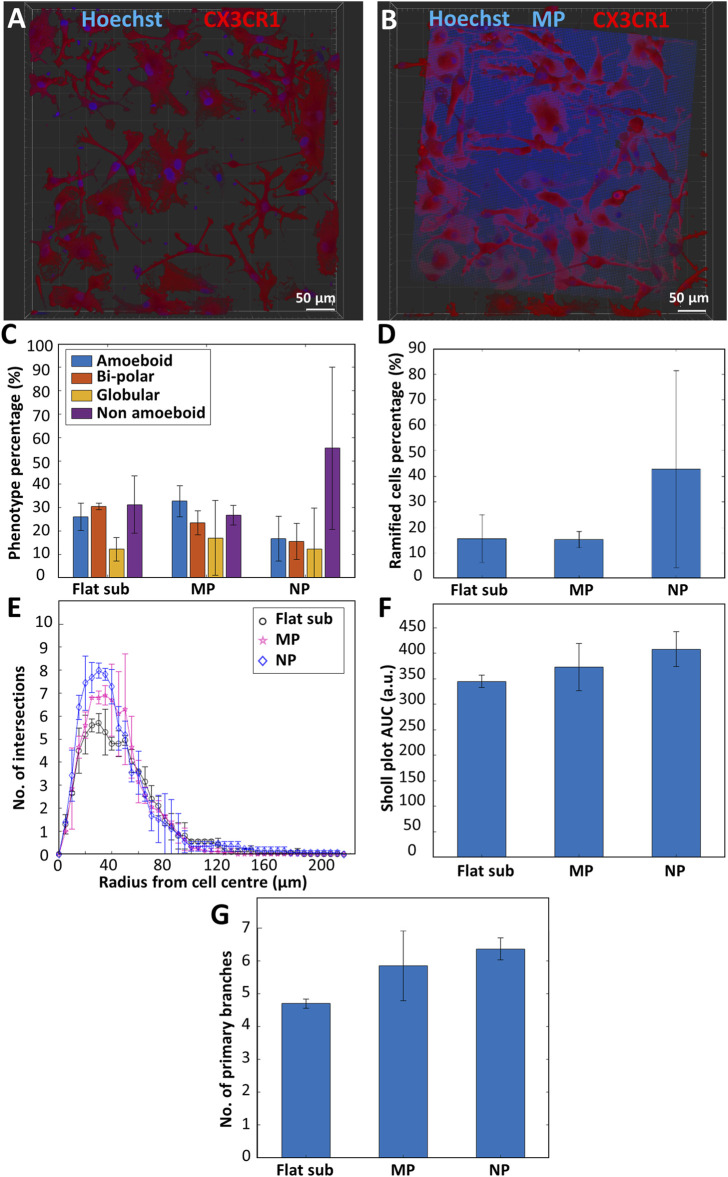
Representative confocal microscopy images of primary microglia (maximum projection) cultured on **(A)** a fused silica substrate; **(B)** micro-pillar arrays. Blue is Hoechst 33342 staining (nucleus) and IP-Dip micro-pillars. Red is CX3CR1 (cell membrane). **(C)** Phenotype distribution of primary microglia cultured on flat fused silica substrates (Flat sub), micro-pillar (MP), and nano-pillar (NP) arrays (n_samples_ = 4 and n_cells_ > 300 for the substrates, n_samples_ = 4 and n_cells_ > 300 for the micro-pillar arrays, n_samples_ = 3 and n_cells_ > 300 for the nano-pillar arrays). **(D)** Percentage of ramified cells. **(E)** Sholl analysis of ramified cells showing the complexity of the cells by indicating the number of intersections the cells have with the concentric contours. **(F)** Area under the curve (AUC) calculated from the Sholl plot. Larger AUC corresponds to a more complex ramified cell. **(G)** Number of primary branches per ramified cell (n_samples_ = 3 and n_ramified_cells_ = 15 for the flat substrates, n_samples_ = 3 and n_ramified_cells_ = 15 for the micro-pillar arrays, n_samples_ = 3 and n_ramified_cells_ = 13 for the nano-pillar arrays).

In order to investigate the effect of surface topography on the expression of a ramified resting phenotype in microglia, we performed *in vitro* experiments comparing the phenotype and morphology of primary microglia cultured on flat fused silica substrates, micro-pillar arrays, and nano-pillar arrays. Figures 5A,B show representative confocal images of microglia cultured on flat substrates and micro-pillars respectively. The high autofluorescence of IP-Dip specifically in the blue channel can be clearly noticed in Figure 5B. Neither flat substrates nor 2.5D-3D structures were coated with laminin in these experiments in light of the observations reported in Section 3.4. A total of two donors were used for this study with 1-3 samples per donor. The results showed a clear difference in phenotypic differentiation when culturing microglia on nano-pillar arrays as compared to micro-pillars or flat substrates (n_samples_ = 4 and n_cells_ > 300 for the substrates, n_samples_ = 4 and n_cells_ > 300 for the micro-pillar arrays, n_samples_ = 3 and n_cells_ > 300 for the nano-pillar arrays). While the percentage of non-amoeboid cells on flat substrates was 31.3 ± 12.3% and on micro-pillar arrays was 26.8 ± 4.14%, it increased to 58.4 ± 34.7% on the nano-pillar arrays. On the other hand, the flat amoeboid phenotype percentage decreased from 26.1 ± 5.9% on the flat substrate and 32.7 ± 6.7% on the micro-pillar arrays to 16.8 ± 9.56% on the nano-pillar arrays. The percentage of the bi-polar phenotype showed a consistent decline also since the percentage was 30.5 ± 1.34% on the flat substrate, 23.4 ± 5.1% on the micro-pillar arrays, and 15.5 ± 7.76% on the nano-pillar ones. Finally, the percentage of globular phenotype was rather consistent on all substrates as its percentage was 12.3 ± 5.03% on the flat substrate, 17.1 ± 15.9% on the micro-pillar arrays, and 12.3 ± 17.4% on the nano-pillar arrays (Figure 5C).

Moreover, Figure 5D illustrates that the ramified phenotype is on average overexpressed when using nano-pillars compared to flat substrates or micro-pillar arrays. A total of 42.8 ± 38.4% of cells cultured on nano-pillar arrays were ramified (approximately 73.3% of non-amoeboid cells). On the other hand, only 15.6 ± 9.31% of cells on flat substrates (approximately 49.8% of non-amoeboid cells) and 15.2 ± 3.2% of cells on micro-pillar arrays (approximately 56.7% of non-amoeboid cells) expressed a ramified morphology. As a quantitative measure of the degree of complexity of the cells (i.e., the branching degree of the cell), Sholl analysis was performed. Briefly, this analysis is based on the counting of the intersections of the body and branches of the cell with concentric contour lines that have a common origin at the center of the cell. The result is displayed as the distance between an intersection and the cell center. In the Sholl analysis (Figure 5E), we show that ramified cells grown on the flat substrate are the least complex cells with the fewest number of branches, followed by ramified cells cultured on the micro-pillars. The cells cultured on the nano-pillars are the most complex, indicated by the highest amount of intersections. This is also confirmed by the AUC values extracted from the Sholl plot. The AUC value for ramified cells cultured on the flat substrate was 345 ± 12.4 arbitrary units (a.u.), while the values for those cultured on micro-pillars and nano-pillars were 373 ± 46.0 a.u. and 408 ± 33.9 a.u. respectively (Figure 5F). In terms of primary branches per ramified cell, a similar upward trend is reported in Figure 5G. The graph shows an average of 4.70 ± 0.14 branches/cell for cells cultured on flat substrates. The number of branches is higher for ramified cells cultured on micro-pillar arrays showing 5.85 ± 1.06 branches/cell. Ramified cells cultured on nano-pillar arrays had on average 6.36 ± 0.34 branches/cell (n_samples_ = 3 and n_ramified_cells_ = 15 for the flat substrates, n_samples_ = 3 and n_ramified_cells_ = 15 for the micro-pillar arrays, n_samples_ = 3 and n_ramified_cells_ = 13 for the nano-pillar arrays). More information about the Sholl analysis, AUC, and primary branches counting can be found in Section 2.

Concerning the high standard deviation in our results, we would like to underline the exploratory nature of our work, which was primarily aimed at investigating if and how micro- and nano-structures could affect microglial morphological features. It is important to mention that technical and practical considerations, pertaining to the production times of micro- and nanostructures and to the availability of tissue donors to initiate primary cell cultures, impede repetitive use of micro- and nanostructures in donor numbers required to obtain statistical significance. In addition, the tissue donors come from an outbred colony of non-human primates, further contributing to increased donor-donor variability.

**FIGURE 6:**
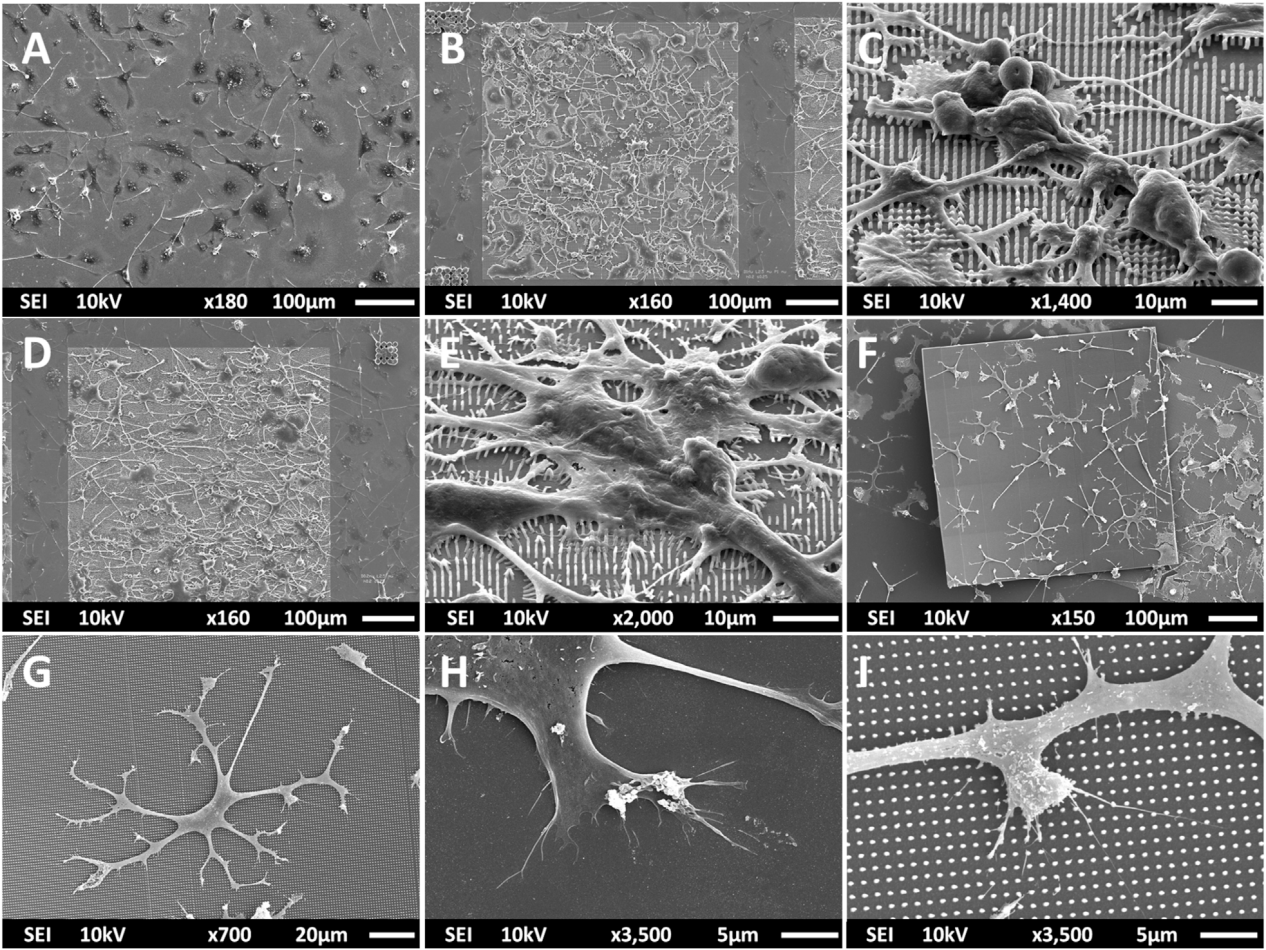
SEM images of primary microglia on: **(A)** a flat fused silica substrate; **(B,C)** micro-pillar arrays; **(D,E)** NP1 nano-pillar arrays (pillars printed directly on the substrate); **(F,G)** NP2 nano-pillar arrays (pillars printed on a pedestal). Ramified microglia were noticed on both NP patterns, but pillars of NP2 had higher structural integrity compared to those on NP1 due to the support of the pedestal. Interaction of microglial filopodia with **(H)** the flat substrate; **(I)** nano-pillar arrays (NP2). **(C,E)** images are acquired at a 45° angle.

The observations pointed out in Figure 5 were corroborated by SEM characterization of fixed and dehydrated cells that showed the difference in microglial phenotype and morphology when cultured on a flat substrate (Figure 6A), micro-pillar arrays (Figures 6B,C), and nano-pillar arrays (Figures 6D–G).

On flat substrates, an abundance in the amoeboid phenotype was noticed in contrast to micro- and nano-pillar arrays. An increased number of ramified cells and primary branches per cell were noticed on the pillar arrays. Overall, no phenotypic differences were noticed between microglia cultured on NP1 (Figures 6D,E) and those cultured on NP2 (Figures 6F,G). The processes of the ramified cells showed strong adhesion and wrapping of the pillars, especially in the nano-pillars configuration. In some cases, the forces applied on the pillars were strong enough to detach them. This was particularly observed in NP1 patterns (Figure 6E). The introduction of a supporting pedestal in NP2 patterns however improved the stability of the nano-pillars (Figure 6G showing a highly ramified cell). In addition to the extensive branching of microglia noticed on the micro- and nano-pillar arrays, a plethora of connections between the cells were also observed alluding to the positive effect of these patterns on the intercellular communication and network formation (Supplementary Figure S6). The interaction between the filopodia and the different structures was evident. We observed that the membrane at the end of the primary branch of the ramified microglia on the flat fused silica substrate is flatter and more spread out (Figure 6H) as compared to the membrane on the branches of the ramified microglia cultured on the micro- and nano-pillar arrays (Figure 6I). The filopodia on the pillar arrays seem to only extend from pillar to pillar and avoid attaching to the fused silica substrate that lies underneath. Therefore, we hypothesize that the pillars have a guidance effect on the filopodia and hence on the direction of growth of the microglial branches.

The larger number of ramified microglia, their primary processes and the noticeable interaction of filopodia with the structures for the cells cultured on micro- and, especially, nano-pillars can possibly be explained by the low effective shear modulus of the arrays and the high number of anchoring points, providing better support for the cells to exert forces on. The diameters of the pillars were in the range of 300 nm to 1 µm which is close to the range of diameters of filopodia (100–300 nm) (Mattila and Lappalainen, 2008), thereby inducing an efficient interaction with them which associate to the preferential growth of ramified cells with multiple protrusions. In addition, it is also hypothesized that the discrete geometry of micro- and nano-pillar arrays provides a biomimetic environment for the microglia since the pointed heads of the pillars resemble the multitude of the micro- and nanometric intersections found in the ECM as a result of the elaborate network of proteins from which the ECM is made. Such protein filaments have diameters in the range of 9–300 nm (Kim et al., 2018; Antonova et al., 2021). Focal adhesions formed on these discretized intersections in the ECM are usually smaller and much scarcer than those formed on 2D flat substrates as shown for mesenchymal stem cells among other cell types in 3D environments (Fraley et al., 2010; Zonderland et al., 2020; Zonderland and Moroni, 2021). As mentioned by Kim et al., it is thought that culturing microglia on structures of similar size ranges as the proteins of the ECM reduces inflammatory responses of the cells since the mechanical cues presented to them are somewhat similar to their native *in vivo* environments (Kim et al., 2018). These biomimetic structures are thought to stimulate the production of certain proteins and transcription factors that aid cell adhesion and define the morphology and phenotype of cells (Schulte et al., 2016; Selvakumaran et al., 2008; Yang et al., 2013; Yang et al., 2015). The effective shear modulus (0.25–14.63 MPa) of the pillars which is closer to the Young’s modulus of brain tissue (0.1–1 kPa) (Lu et al., 2006) compared to the bulk Young’s modulus of the fused silica substrate (∼72 GPa) (Torres-Torres et al., 2010) also plays a fundamental role in the increase of ramified microglia. Based on our results, we conclude that the lower the effective shear modulus, the more ramified microglia are present and the higher the degree of ramification of these cells is observed.

Our results are supported by other studies, performed on non-primate derived cells, that utilized a nanotopography to stimulate the growth of a ramified microglial phenotype resembling microglia *in vivo* (Chen et al., 2010; Pires et al., 2015; Song et al., 2020, Song et al., 2014). In the study conducted by Song et al., modified bacterial cellulose nanofibril substrates were shown to increase the complexity of primary rat microglia (i.e., the degree of ramification) by a factor of 1.7 when compared to cells cultured on flat glass substrates (Song et al., 2020). Pires et al. showed in their study an increase in microglial ramification upon culturing primary rat microglia on poly (trimethylene carbonate-co-1-caprolactone) electrospun fibers of 200 nm–2 µm diameters (Pires et al., 2015). When it comes to the effect of stiffness on the morphology of microglia however, the literature shows some contradiction. In a study performed by Blaschke et al., rat primary microglia were shown to a have less round and more polar morphology and express more anti-inflammatory cytokines when cultured on a soft polydimethylsiloxane (PDMS) substrate (Young’s modulus = 0.6 kPa) versus a stiffer one (Young’s modulus = 1.2 MPa) (Blaschke et al., 2020). On the other hand, Dudiki et al. showed in their study that when culturing murine primary microglia on fibronectin-coated hyaluronic acid-based hydrogels of 60 and 600 Pa Young’s moduli, the bipolarization of the cells increased by approximately 3 times on the stiffer hydrogel (Dudiki et al., 2020). Nouri-Goushki et al. emphasized the importance of effective shear modulus when showing the positive effect of the height of nano-pillar arrays on polarization of murine macrophages (Nouri-Goushki et al., 2021).

Taken together, our results suggest that the resemblance of our 2.5D nano-pillar arrays to the adhesion discrete sites in 3D environments and the more biomimetic effective shear modulus of these arrays, affects the formation and sizes of focal adhesions and hence the morphology and phenotypic expression of primary microglia derived from adult rhesus macaques. It should be noted that the interplay between the effective shear modulus and the topography of the pillar arrays as well as the effect of each of them on the morphology of microglia should be further investigated. It is not possible to claim one or the other as the main reason for the increased number of the analyzed ramified microglia cells.

### 3.6 Culture of Microglia in 3D Polymeric Scaffolds

**FIGURE 7:**
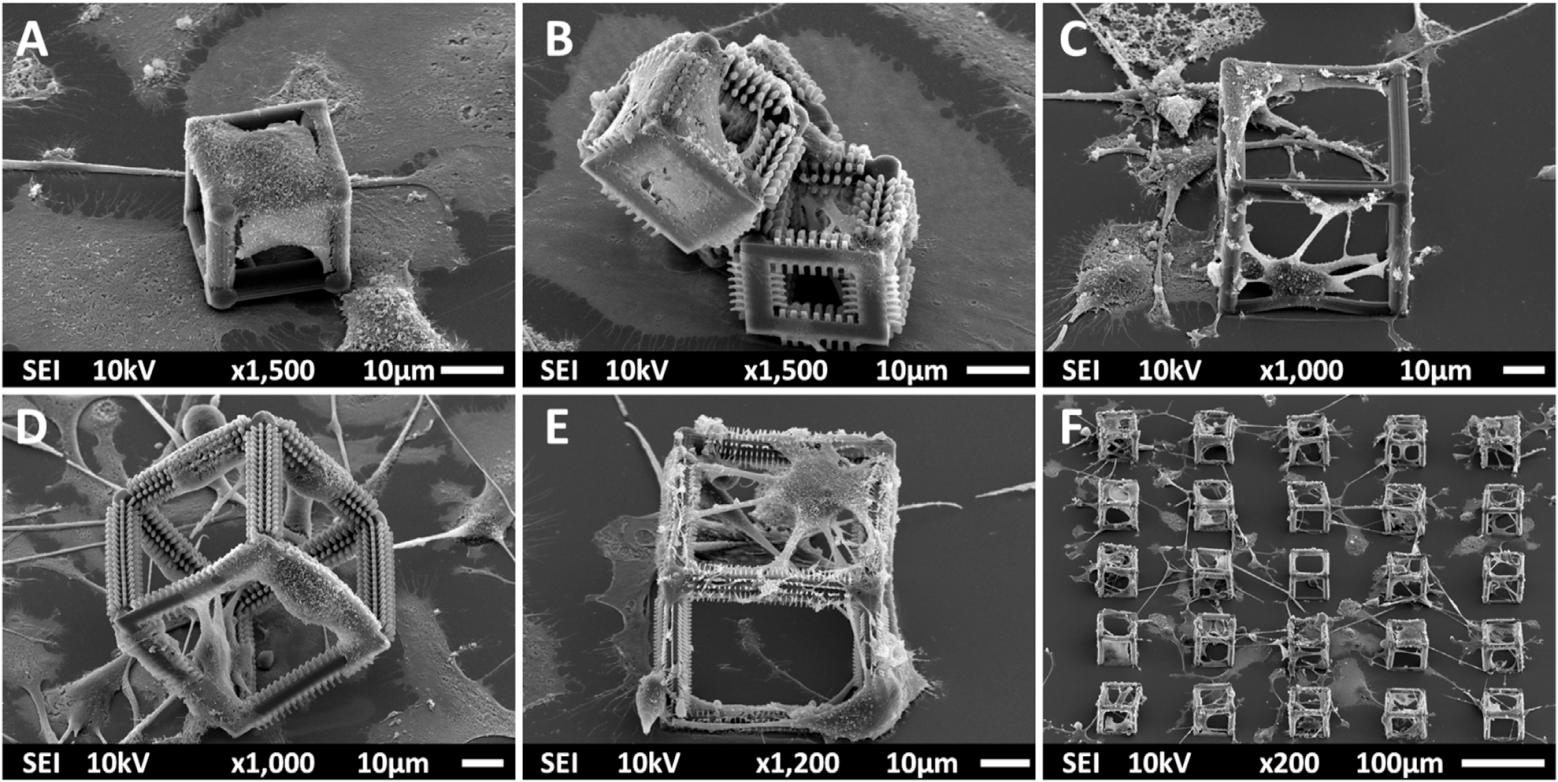
SEM images (taken at a 45° angle) of primary microglia cultured on **(A)** SC; **(B)** SC-MP; **(C)** BC; **(D)** BC-MP; **(E)** and **(F)** BC-NP. An amoeboid-like morphology of microglia was observed when cultured on SC and SC-MP. Cells seemed to attempt to enwrap the cages. In presence of the bigger cages, multiple phenotypes were noticed. Detachment of the cages was observed especially for the smaller ones.

To provide a biofidelic environment for primary microglia cultured *in vitro*, 3D polymeric scaffolds were fabricated by 2PP at different scales. Microglia were cultured on undecorated 25 × 25 × 25 μm^3^ small cages, small micro-pillar decorated cages, 50 × 50 × 50 μm^3^ big cages, micro-pillar decorated big cages, and finally nano-pillar decorated big cages (Figure 7). SEM characterization studies were performed on microglia from three donors of which 1 was used for cell scaffold-occupancy assessment. An interesting discrepancy in the behavior of microglia cultured on small versus big cages was qualitatively observed. Microglia displayed a flat amoeboid-like morphology in presence of the SC (Figure 7A) and SC-MP cages (Figure 7B) and tried to enwrap them in a manner very reminiscent of phagocytosis. In addition, detachment of multiple small cages was noticed which may have been caused by the forces applied by microglia on the cages. When cultured on big cages, the cells wrapped around single beams of BC (Figure 7C), BC-MP (Figure 7D), and BC-NP (Figures 7E,F) showing multiple phenotypes and extending their processes from one end to the other within each cage.

The phagocytosis of the SC and SC-MP may be explained by the fact that the size of the amoeboid phenotype of the microglia used in this study was measured to be roughly 40 µm which means that the area of this phenotype is larger than that of SC, but smaller than that of BC. Previous works also show that the current predominant opinion regarding the sizes of foreign bodies that can be phagocytosed by a single macrophage is between 500 nm and 10 µm (Xia and Triffitt, 2006; Mitragotri and Lahann, 2009). Bodies larger than 10 µm but smaller than 100 µm may also be phagocytosed and digested by large assemblies of macrophages referred to as foreign body giant cells. Another possibility however is that macrophages may adhere to these relatively larger bodies and try to degrade them by producing multiple enzymes. It is not clear, however, why the microglia did not form bigger agglomerates to attempt phagocytosis of the BC and its variants. One possible reason is the large area and volume of the cages that hindered the cells from wholly engulfing them. Therefore, they wrapped around single beams instead. As for the other cell morphologies identified in the bigger cages, a possible explanation may be the increased points of contact provided by both the 3D geometry of the structure and the micro- and nano-pillars decoration. This hypothesis is supported by the study of Tylek et al. in which human monocyte-derived macrophages were cultured on poly (ε-caprolactone) micro-fibers with varying pore sizes (40–100 µm) and showed increased polarization in the smaller pores (Tylek et al., 2020). It has to be noted though that the difference in material, stiffness, and cell type must be kept in mind when making such comparisons. In addition, there is some inconsistency in literature regarding the effect of pore sizes on macrophages as it has been shown in other studies that larger pore sizes induce a more polarized morphology of macrophages while smaller ones confine them to a round shape (Liang et al., 2018; Jiang et al., 2019). In light of this, further studies focusing on the effect of a softer scaffold with a similar geometry on the phenotype of microglia would be needed.

**FIGURE 8:**
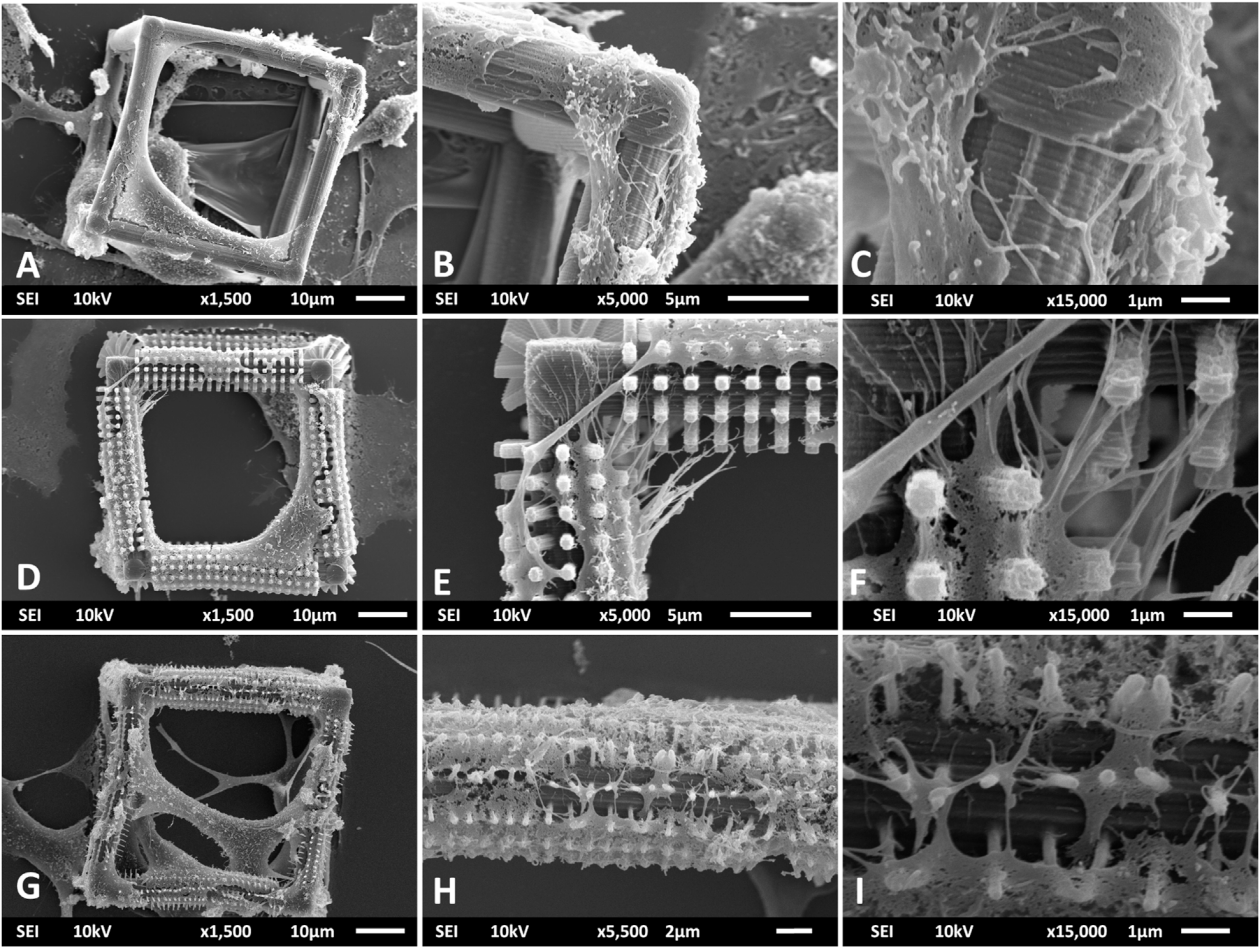
SEM images showing the interaction of primary microglia and their filopodia with the micro- and nanometric features of the BC **(A–C)**, BC-MP **(D–F)**, and BC-NP **(G–I)**. **(C)** Filopodia guided by the nano-grooves formed by the hatching lines in the BC. **(F)** Micro-pillars acting as a competing guidance cue to the filopodia. **(I)** Nano-pillars providing a mechanical cue and a platform of growth for the cells.

An additional investigation of the interaction of filopodia with the micro- and nano-features of the cages was performed using SEM imaging (Figure 8). The filopodia mostly extend in multiple directions with respect to the hatching lines of the undecorated BC (Figures 8A–C). The most predominant direction is along the hatching lines (Figure 8C). This indicates that these lines act as topographical guidance cues to the filopodia. A possible reason for such a preference in growth direction can be the proximity of the size of hatching lines (hatching distance = 200 nm) to that of filopodia (Mattila and Lappalainen, 2008). Another possible reason is the confinement of focal adhesions to these nanometric ridges (Tonazzini et al., 2020). Similar results were found in multiple studies such as that of Fujita et al. where filopodia of mesenchymal stem cells showed a preference towards extending along nanometric ridges instead of perpendicular to them (Fujita et al., 2009). In that study, they also pointed that the formation of longer and more stable focal adhesions in the longitudinal direction could be one of the main reasons why the filopodia chose to grow specifically in that direction. An interesting change was observed in the interaction of microglia and their filopodia with the pillars on the beams of BC-MP (Figures 8D–F). The filopodia on the BC-MP extend in multiple directions bridging distances between pillars and do not strictly follow the hatching lines on the cage especially in regions where there is a high density of micro-pillars (Figures 8E,F). This can be explained by the fact that the micro-pillars represent a competing guidance cue for the filopodia, specifically, providing an anchor for the movement of the cells. This observation suggests that filopodia, once in contact with the pillars, specifically choose to refrain from interacting with the undecorated surface. When cultured on the BC-NP structure, microglia exhibited an increased interaction with the nano-pillars (Figures 8G–I). Once again, in high density regions of pillars, the membranes of the cells stretched mainly over the pillars with little to no interaction with the underlying hatching lines (Figures 8H,I) proving that these pillars indeed represent a mechanical cue involved in the guidance and growth of the cells. What seems to be membrane rupture was noticed however when the cells wrapped around the nano-pillars decorating the beams of BC-NP (Figure 8I) which is a known problem that is thought to occur due to the dehydration step performed during the preparation of the samples for SEM imaging (Lee and Chow, 2012; Jusman et al., 2014; Katsen-Globa et al., 2016). These results show that our HMDS protocol was only partially successful in preventing membrane rupture. Further optimization of the dehydration of cells should be carried out in future studies to completely eliminate this undesirable effect. An alternative explanation of the observation in Figure 8I is that these extensions maybe filopodia wrapping around single nano-pillars.

**FIGURE 9:**
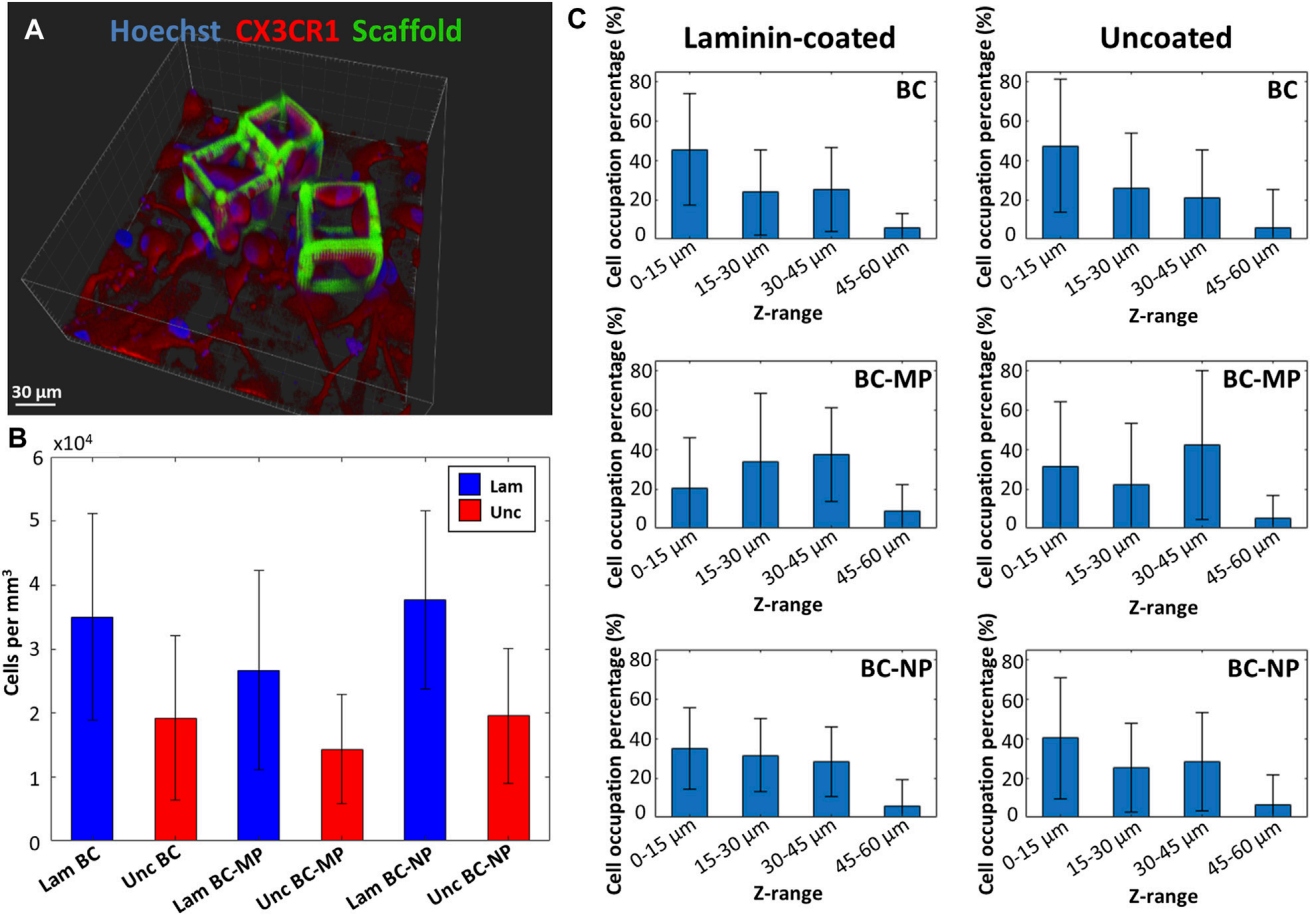
**(A)** 3D reconstruction of a fluorescence z-stack obtained by confocal microscopy of BC-MP cages with microglia. Blue is Hoechst 33342 staining (nucleus) and red is CX3CR1 (cell membrane). **(B)** Volumetric occupancy of microglia in laminin-coated (blue) and uncoated (red) cages. Laminin coating resulted in doubling the occupancy of cages as compared to uncoated ones for decorated and undecorated cages alike. **(C)** Microglia distribution along the height of the BC, BC-MP, and BC-NP for laminin-coated and uncoated samples. Microglia were shown to colonize the mid and top sections of decorated structures compared to the undecorated ones (for each structure type n_samples_ = 2, n_cages_ > 25, n_cells_ > 100).

The overall degree of occupation of cells within the bigger cages was measured as well using z-stacks of the fluorescence images obtained by confocal microscopy (Figure 9A). It was expressed in terms of occupied volume of cells per cage (Figure 9B) (for each structure type n_samples_ = 2, n_cages_ > 25, n_cells_ > 100). The results show an increase in colonisation of the cages when laminin is used as a coating. On average, laminin-coated cages had twice as many cells as compared to uncoated cages. More specifically, the laminin-coated BC had on average 3.5 ± 1.61 × 10^4^ cells/mm^3^, which is higher than the uncoated BC which had on average 1.92 ± 1.28 × 10^4^ cells/mm^3^. The laminin coated BC-MP had on average 2.67 ± 1.56 × 10^4^ cells/mm^3^ which was also higher than the uncoated BC-MP 1.43 ± 8.52 × 10^4^ cells/mm^3^. The laminin-coated BC-NP had 3.77 ± 1.39 × 10^4^ cells/mm^3^ while the uncoated BC-NP had 1.96 ± 1.06 × 10^4^ cells/mm^3^. This positive effect of laminin coating on the colonization of the cages maybe explained by the added resemblance to the ECM that such a biochemical coating provide since laminin is a major component in the ECM of the CNS (Kim et al., 2018). The structures present a large surface area with many micro- and nanometric features, grooves, and crevices consisting of micro- and nano-pillars in addition to hatching lines and slices produced by the 2PP process. We hypothesize that this surface area, acting as a basin, can be the reason for the accumulation of laminin on these structures, thereby attracting microglia to colonize them. In an attempt to quantify the interaction of microglia with the 3D bigger cages, we investigated which region of the cages were most densely populated (for each structure type n_samples_ = 2, n_cages_ > 25, n_cells_ > 100). The cages were split up into four sections depending on their height, namely 0–15, 15–30, 30–45, and 45–60 µm. The degree of cell colonization is expressed as the percentage of microglia occupying each section of the cages (Figure 9C). A comparison between laminin-coated cages and uncoated cages is presented in the figure as well. It can be clearly seen that the percentage of microglia situated at the bottom of the cages was higher for undecorated cages regardless of the coating with laminin. For pillar-decorated cages, and especially micro-pillar-decorated ones, the distribution of cells throughout the height of the cages was more uniform. Even though this part of the study was performed using cells from only one donor, a clear difference can be seen in the way cells interact with and migrate up the beams of the decorated cages. It appears that providing the cells with anchor points in the form of pillars increases their possible points of contact and hence facilitates migrating towards the upper regions of the cages. It can also be argued that since the micro-pillars are larger and less fragile than the nano-pillars, they would provide more stable anchor points for the cells. These results are in line with those of Leclech et al. where neurons where shown to migrate faster in an *in vitro* environment of micro-pillars (Leclech et al., 2019). Bugnicourt et al. also showed how neurites of murine hippocampal neurons featured faster growth when cultured on nano-pillars (Bugnicourt et al., 2014).

## 4 Conclusion

In the current study, we proposed the fabrication of 2.5D micro- and nano-pillars and 3D micro-cages to study the effects of engineered microenvironments on the phenotype of primary microglia derived from brain tissue of adult primates (rhesus macaques). The arrays of pillars were manufactured using 2PP technology with a stiff thermosetting polymer. They have the advantage of being easily fabricated due to the high stiffness of the material while providing a surface with a low effective shear modulus that better resembles the stiffness of brain tissue. In addition, they provide a discrete type of topography that is thought to also mimic that of the ECM and hold dimensions in the range of filopodia processes.

For the first time, we report how primate-derived primary microglia cultured on nano-pillar arrays, show on average an increase in the numbers of cells characterized by a ramified resting phenotype as compared to cells cultured on flat stiff substrates. This ramified morphology is associated with a homeostatic phenotype. However, further gene or protein expression analysis should be carried out to confirm the homeostatic function of these cells. Optimization of our experimental settings by e.g., the integration of a bio-inert coating on the substrate enabling the cells to adhere only on the 2.5D and 3D structures might allow for functional and gene expression analyses (which would be otherwise polluted by input derived from cells adhering on the flat substrate). Regarding morphological features, it is important to note that the percentage of the flat amoeboid phenotype also simultaneously decreased in presence of micro- and nano-pillars. Moreover, we fabricated 3D micro-cages, decorated with micro- or nano-pillars, and employed them as scaffolds in order to emulate the three-dimensional spatial configuration of the native environment of microglia cells. Smaller cages were shown to be enwrapped by microglia, while cages of larger size than the average size of the cell body promoted the expression of multiple microglia phenotypes. Interestingly, cell colonization was affected by both laminin coating, which increased cellular occupation of the cages, and pillar decoration of the beams of the cages. Cells cultured on micro-pillar decorated cages especially showed a higher affinity to occupy the mid and upper sections of the cages suggesting a strong interaction between the microglia filopodia and the pillars. Future studies will be conducted to disentangle the effect of topography from that of substrate stiffness on the morphology and phenotypic expression of microglia. Additionally, it will be important to use our approach in presence of primary microglia from different species and compare the obtained results with the ones reported here. In conclusion, the proposed approach paves the way for a series of investigations, which can include co-culture studies involving microglia and other neuronal lineages in both healthy and diseased states.

## Data Availability

The data that support the findings of this study are available from the corresponding author upon reasonable request.
