# Supplementary material for: Two-Photon Polymerization of 2.5D and 3D Microstructures Fostering a Ramified Resting Phenotype in Primary Microglia
Source: Front Bioeng Biotechnol. 2022 Jul 22;10:926642. doi: 10.3389/fbioe.2022.926642 (PMC9376863; doi:10.3389/fbioe.2022.926642)
Supplement: Supplementary file 1 [file DataSheet1.pdf]

Supplementary Material

Two-photon polymerization of 2.5D and 3D microstructures fostering a ramified resting phenotype in primary microglia

Ahmed Sharaf[1†], Brian Roos[1†], Raissa Timmerman[2], Gert-Jan Kremers[3], Jeffrey Bajramovic[2], Angelo Accardo[1*]

[1]Department of Precision and Microsystems Engineering, Delft University of Technology, Mekelweg 2, 2628 CD, Delft, the Netherlands

[2]Alternatives Unit, Biomedical Primate Research Centre, Lange Kleiweg 161, 2288 GJ, Rijswijk, the Netherlands

[3]Erasmus Optical Imaging Centre, Erasmus MC, Dr. Molewaterplein 50, 3015 GE Rotterdam, the Netherlands

*** Correspondence:**
Angelo Accardo
A.Accardo@tudelft.nl

[†]These authors have contributed equally to this work and share first authorship

3D CAD models of a pedestal, a pillar array, and a pillar-decorated cage.

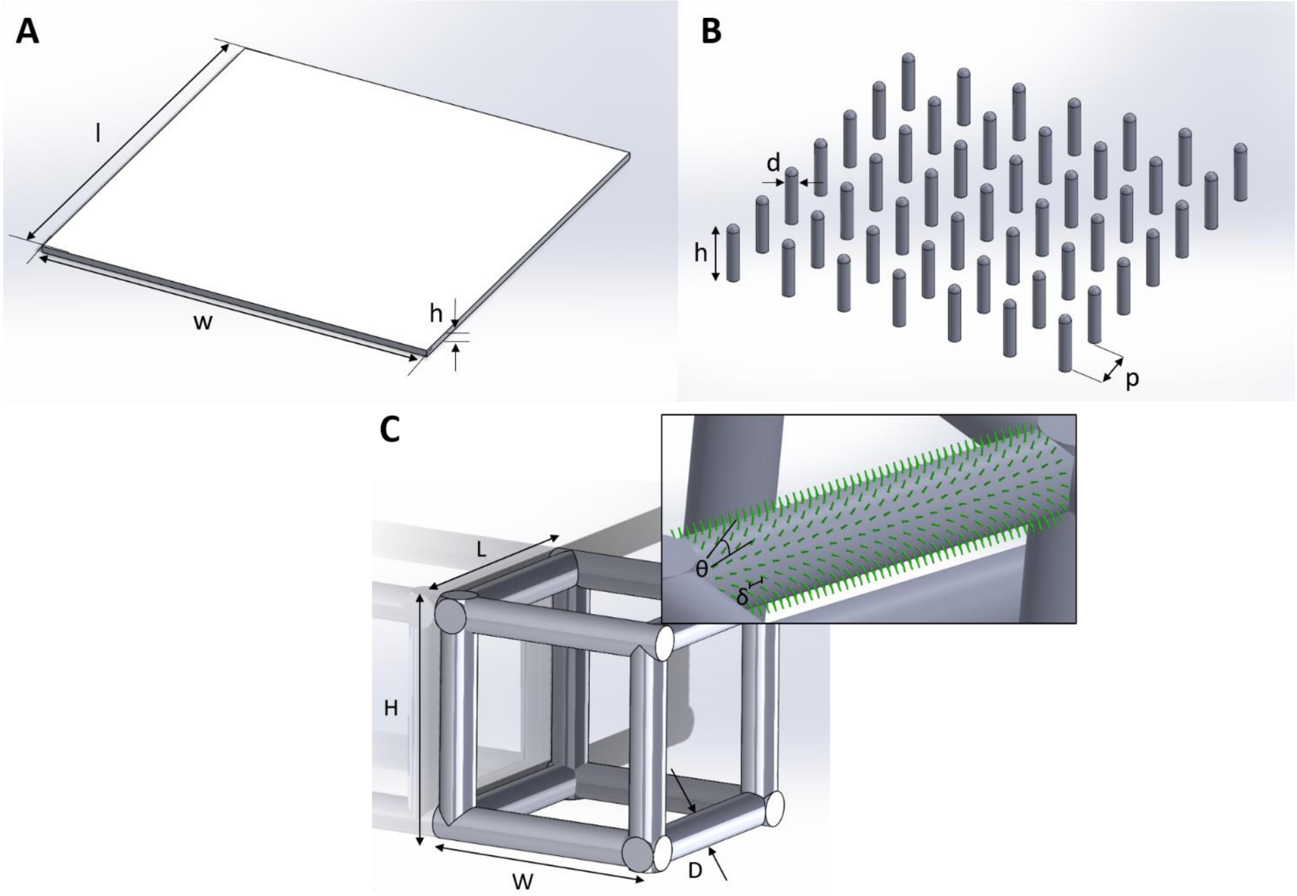

Supplementary Figure 1. 3D CAD models of 2D, 2.5D, and 3D structures created by SOLIDWORKS (DASSAULT SYSTEMES). **(A)** Pedestal (2D) design. l is the length, h is the height and w is the width. **(B)** 2.5D pillar array design. d, h and p are the pillar diameter, height and spacing between pillars, respectively. **(C)** 3D CAD model of a cage design with pillar decoration. H represents the height, W represents the width, L represents the length, and D represents the beam diameter. For the pillar decorations, θ = angular spacing between pillars and δ = lateral spacing between pillars.

Schematic representation of mechanical strength characterization using FEMTOTOOLS nanomechanical testing system FT-NMT03 and AFM.

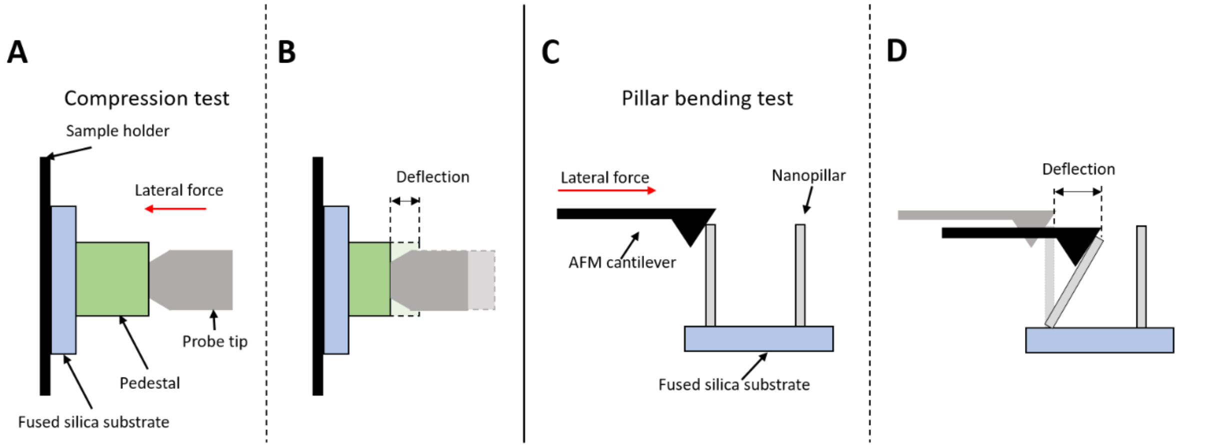

Supplementary Figure 2. (A), (B) Schematic of the compression test using the Femtotools. **(A)** Femtotools probe tip applies a lateral force on the pedestal. **(B)** Deflection of pedestal caused by the applied force of the tip of the probe. **(C), (D)** Schematic of the AFM pillar bending test. **(C)** AFM probe tip applies a lateral force on the nano-pillar. **(D)** Deflection of nano-pillar caused by the applied force of the tip of the AFM.

SEM characterization of micro-pillars and nano-pillars on cuboidal cages.

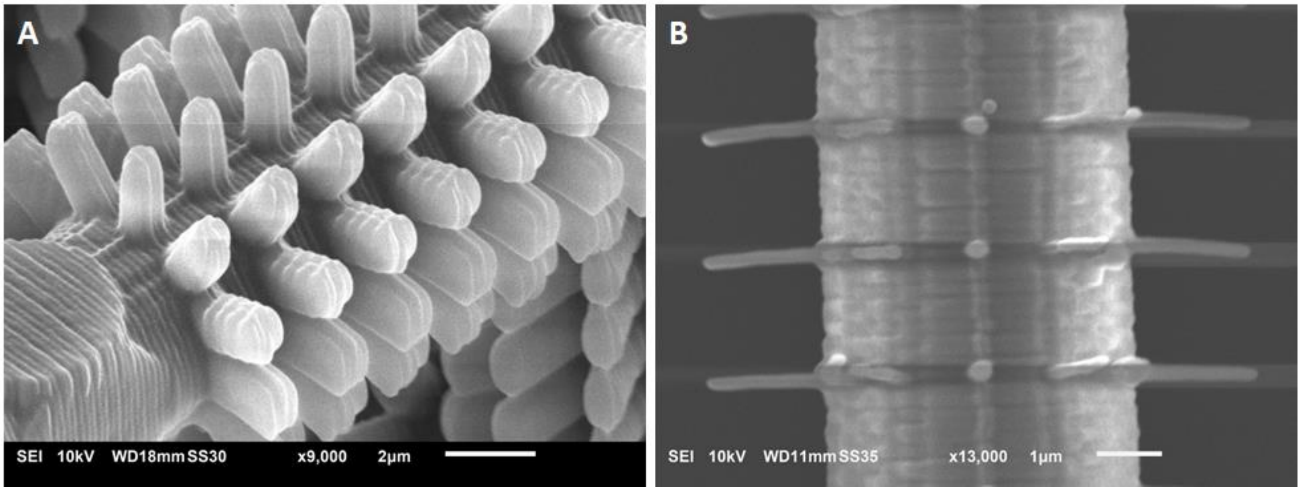

Supplementary Figure 3. SEM micrograph of the micro- **(A)** and nano-pillars **(B)** decorating the cuboidal cages.

***In vitro* classification of primary microglia.**

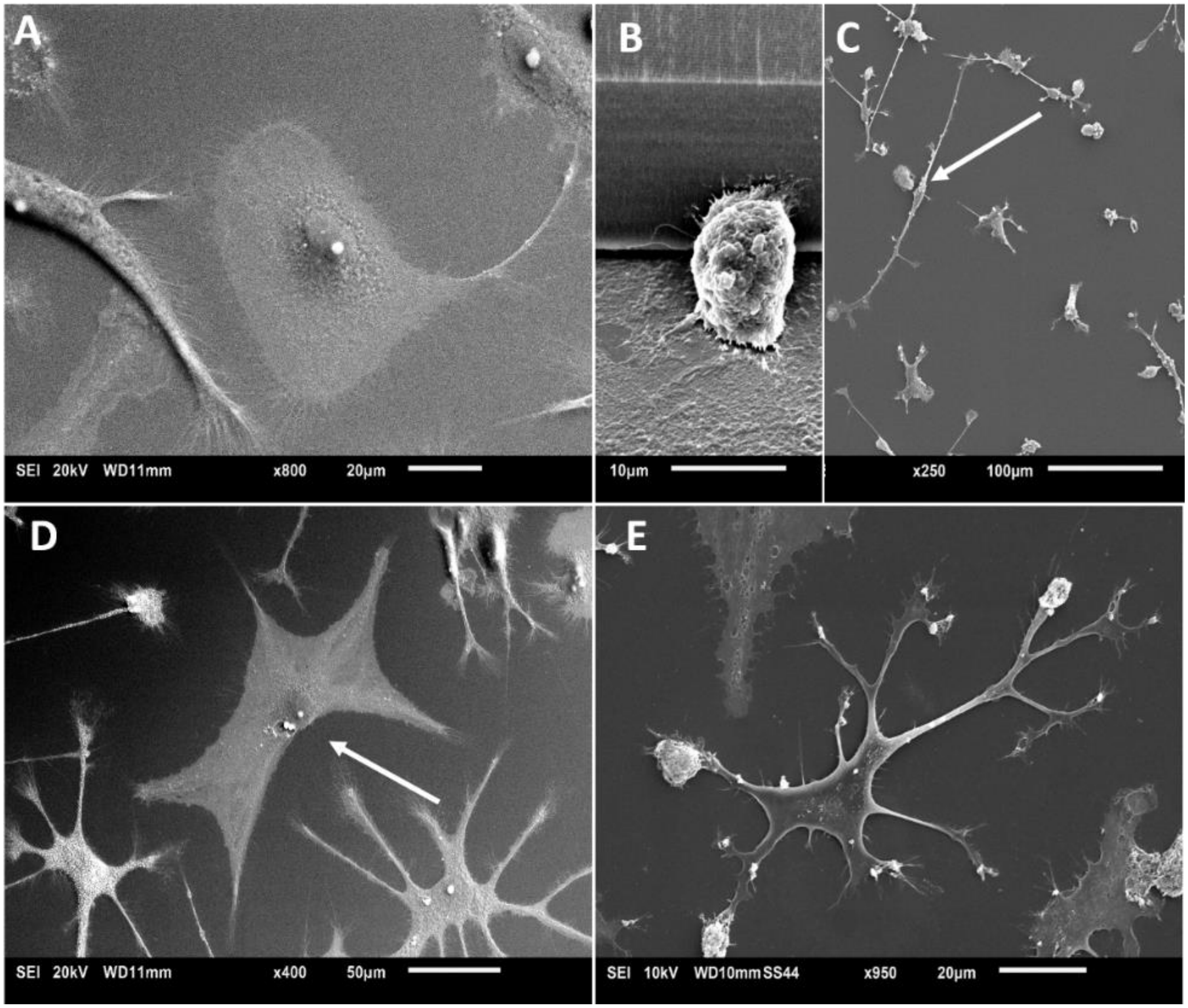

Supplementary Figure 4. SEM images of the phenotypes of primary microglia *in vitro*. **(A)** Flat amoeboid cell, a round cell with a flattened cell body. **(B)** Globular cell, spherically shaped cell with a small soma. **(C)** Bi-polar phenotype is a long rod-shaped cell with two poles extending from each side of the soma (white arrow). **(D), (E)** Non-amoeboid phenotype. **(D)** Non-ramified cell with short underdeveloped branches (white arrow). **(E)** Ramified phenotype, showing 6 primary branches with side branches and a relatively small soma.

Effect of laminin on the phenotypic expression of primary microglia.

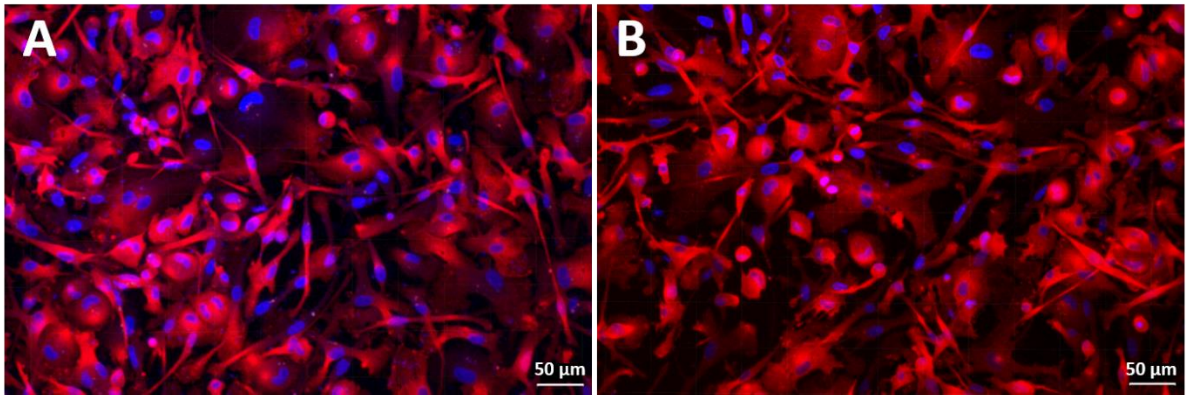

Supplementary Figure 5. Representative confocal microscopy images of primary microglia on **(A)** an uncoated fused silica substrate and **(B)** laminin-coated substrate.

Effect of micro- and nano-pillar arrays on the phenotype, morphology and network formation of primary microglia.

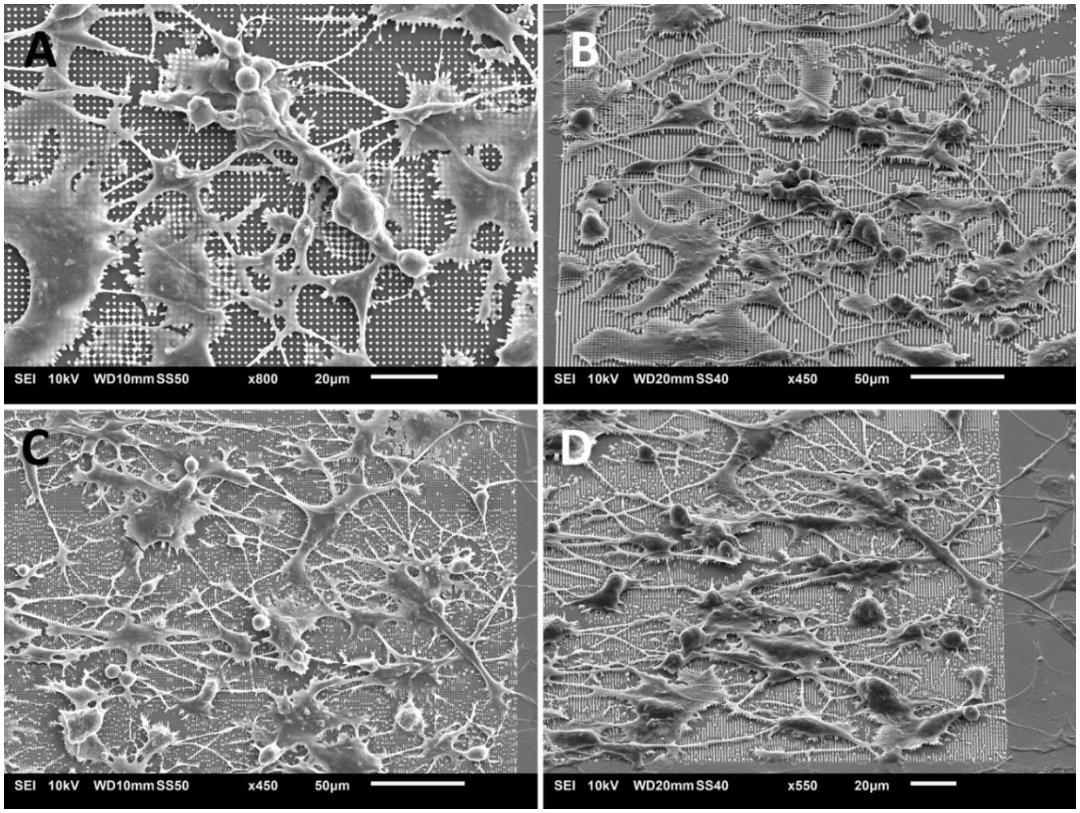

Supplementary Figure 6. Representative SEM images of primary microglia on **(A)**, **(B)** MP1 micro-pillar arrays and **(C), (D)** NP1 nano-pillar arrays. The images show the heavy interconnection and networks formed between the microglia. B and D are taken at a 45° angle.

Light intensity values

The light intensity values corresponding to the laser power values (35 and 42.5 mW) employed to print the structures were calculated using **Eq. 1** (Skliutas et al., 2021).

$$\boldsymbol{I = \frac{2PT}{R{w_0}^2\pi\tau}} \quad \textbf{(1)}$$

Where I is the light intensity of the laser, P is the laser power, T is the transmittance of the employed objective, R is the pulse repetition rate of the laser, w_0 is the waist radius of the laser beam, and τ is the pulse duration of the laser source.

Supplementary Table 1. Calculated light intensities. All values pertaining to the properties of the laser source were provided by Nanoscribe GmbH & Co. KG.

**Objective transmittance (T)**	0.75	
**Pulse repetition rate (R) [MHz]**	80.15	
**Radius at beam waist (w_0) [nm]**	340	
**Pulse duration (τ) [fs]**	95.3	
**Laser power (P) [mW]**	35	42.5
**Light Intensity (I) [TW/cm^2]**	1.89	2.29

Information of rhesus macaque donors from which primary microglia were isolated.

Supplementary Table 2. Information of rhesus macaques microglia donors.

Donor	Age [year]	Gender	Weight [kg]	Origin
**D1**	13	Female	7.6	India
**D2**	13	Male	10.7	India
**D3**	19	Male	13.1	India
**D4**	3	Male	7.85	India
**D5**	5	Female	4.6	India
**D6**	11	Male	17.4	India

Measured dimensions of the 2D, 2.5D, and 3D structures.

Supplementary Table 3. Measured dimensions of 2D and 2.5D structures.

Measured dimensions	Pedestal	MP1	MP2	NP1	NP2
Width (w) [μm]	121±1.07	-	-	-	-
Diameter (d) [μm]	-	0.97±0.04	1.06±0.07	0.39±0.02	0.29±0.01
Height (h) [μm]	19.4±0.36	1.97±0.04	1.87±0.05	1.90±0.07	1.82±0.06
Spacing (p) [μm]	-	1.01±0.02	0.97±0.05	0.83±0.04	0.76±0.03

Supplementary Table 4. Measured dimensions of 3D structures.

Measured dimensions	SC	SC-MP	BC	BC-MP	BC-NP
Width (W) or Length (L) [μm]	24.2±0.09	24.1±0.15	49.0±0.07	46.9±0.44	46.1±0.33
Beam diameter (D) [μm]	4.92±0.05	4.90±0.05	4.98±0.05	4.75±0.06	4.89±0.03
Height (H) [μm]	24.3±0.04	23.7±0.20	48.8±0.11	43.7±0.68	46.0±0.34
Pillar diameter [μm]	-	1.01±0.04	-	0.88±0.05	0.24±0.02
Pillar height [μm]	-	2.20±0.02	-	2.27±0.03	1.86±0.02
Angular spacing (θ) [◦]	-	29.4±0.22	-	30.1±0.52	31.0±0.88
Lateral spacing (δ) [μm]	-	0.97±0.02	-	1.00±0.08	0.83±0.02

Additional considerations on the data analysis

We considered different statistical analytical test methods to handle the large donor-donor variability that we encounter using outbred animals. A standard paired t-test could be used to analyze differences within the same animal, assuming that the distribution of these differences is normal. If one assumes that these differences are not normally distributed, a Welch's test can be considered. Either way, the combination of high standard deviations and low donor numbers precluded meaningful statistical analysis.

References

Skliutas, E., Lebedevaite, M., Kabouraki, E., Baldacchini, T., Ostrauskaite, J., Vamvakaki, M., Farsari, M., Juodkazis, S., Malinauskas, M., 2021. Polymerization mechanisms initiated by spatio-temporally confined light. Nanophotonics 10, 1211–1242. https://doi.org/10.1515/nanoph-2020-0551
